# Improvement and tolerance mechanisms of *Priestia megaterium* to salt ions

**DOI:** 10.3389/fmicb.2025.1730703

**Published:** 2025-12-18

**Authors:** Chunlong Wang, Shaohua Chu, Dan Zhang, Pei Zhou, Yimin You

**Affiliations:** 1 Jilin Provincial Key Laboratory of Tree and Grass Genetics and Breeding, College of Forestry and Grassland Science, Jilin Agricultural University, Changchun, China; 2 School of Agriculture and Biology, Shanghai Jiaotong University, Shanghai, China; 3 Key Laboratory of Urban Agriculture, Ministry of Agriculture and Rural Affairs, Shanghai Yangtze River Delta Eco-Environmental Change and Management Observation and Research Station, Ministry of Science and Technology, Ministry of Education Bor S. Luh Food Safety Research Center, Yunnan Dali Research Institute, Shanghai Jiaotong University, Shanghai, China; 4 Inner Mongolia Academy of Agricultural & Animal Husbandry Sciences, Hohhot, China

**Keywords:** improvement effect, nitrate assimilation pathway, salt stress, secondary salinized soil, tolerance mechanisms

## Abstract

**Introduction:**

Salinity is a major abiotic stress threatening global agriculture. While some microorganisms are known to ameliorate soil salinity and promote plant growth, the underlying mechanisms, particularly for *Priestia megaterium* (formerly *Bacillus megaterium*), remain less explored.

**Methods:**

Here, we investigated the efficacy and mechanism of P. megaterium NCT-2 in improving secondary saline soil by elemental analysis, 15N tracing, gene knockout and transcriptomics.

**Results:**

Our results demonstrated that the NCT-2 agent significantly reduced the content of key salt ions, notably NO₃^−^, Cl^−^, and Na^+^ in soil. Through a combination of biochemical assays, isotope tracing, and gene knockout techniques, we identified that the aerobic assimilation pathway is the primary route for nitrate metabolism in NCT-2, with the *nasC* and *nasD* genes being crucial for this process. Furthermore, transcriptomic analysis under salt stress revealed that NCT-2 employs a multi-faceted tolerance strategy, which includes enhancing sporulation, activating antioxidant defenses (e.g., CAT, SOD), assembling flagella, and forming vesicles. Concurrently, the strain upregulates central carbon metabolism (TCA cycle, glycolysis) and amino acid synthesis to fuel these adaptive responses.

**Discussion:**

This study provides a comprehensive theoretical foundation for using *P. megaterium* NCT-2 in environmental remediation and identifies key genetic targets for enhancing microbial salt tolerance.

## Graphical abstract

**Figure:**
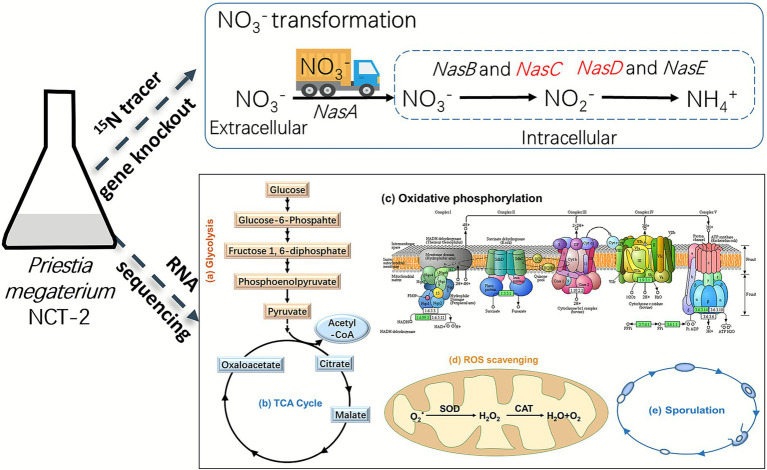

## 1 Introduction

Soil salinity is a serious abiotic stress that influences plant growth and soil productivity all around the globe (Abiala et al., 2018). Salinity induces ionic toxicity, osmotic stress, and mineral deficiency in plants and microorganisms, which increases the technical difficulties of remediation (Xu et al., 2020). Certain rhizospheric bacteria have the potential to promote plant development, augment salt stress, and improve soil quality (Song et al., 2021). Therefore, microbe-assisted remediation is a promising strategy for addressing soil salinity. Moreover, knowing the mechanisms of salt tolerance in microorganisms can reveal several genetic targets for the development of salt-tolerant recombinant bacteria and plants.

Secondary salinized soil, a major threat to global agriculture, refers to the accumulation of water-soluble salts in soil layers due to improper human activities such as excessive irrigation with poor drainage. This process significantly degrades soil health and inhibits plant growth. *Priestia megaterium* (*P. megaterium*, formerly *Bacillus megaterium*) is an important rhizosphere bacterium ubiquitous in the environment. Its application in the remediation of various pollutions has become one of the research hotspots. For example, *P. megaterium* can effectively degrade polycyclic aromatic hydrocarbons, organophosphorus pesticides, dichloroaniline, sulfonamides, and other dangerous substances as a bioremediation agent (Meena et al., 2016; You et al., 2018). *P. megaterium* not only has high metal tolerance, but also can remove metals (Xiao et al., 2021). Furthermore, *P. megaterium* can be used to improve soil salinity, increase nutrients, enhance plant biomass, promote chlorophyll, and antioxidant enzyme activity (Abdel Motaleb et al., 2020). Additionally, *P. megaterium* could increase the production of proline and indoleacetic acid (auxin) in osmotic stress (Marulanda et al., 2009). Therefore, it is certain that *P. megaterium* could resist different abiotic stresses and improve the environment. Thus, studies on the remediation mechanisms and adaptation strategies of *P. megaterium* to salt stress can provide significant theoretical references for the application of this strain to environmental stress.

Current research focuses on the salt environment of secondary salinized soil in greenhouses, including nitrate, sulfate, chloride, sodium, and calcium ions. One of the most important environmental stressors is nitrate (Zhang et al., 2021). Herein, a salt-tolerant strain of P. megaterium NCT-2, which was isolated from salinized soil and shows potential for its remediation, was selected for this study (Chu et al., 2018; You et al., 2021). Based on this, the present study combines isotope labeling, gene knockout, and transcriptomics to explore the mechanisms by which *P. megaterium* NCT-2 reduces salt ion content and tolerates salt stress in secondary saline soil. It is expected that this study will add to our understanding of *P. megaterium’s* resilience to salt stress and shed light on the development of microbial agents and the role of rhizosphere bacteria in abiotic stress remediation.

## 2 Materials and methods

### 2.1 *Priestia megaterium* NCT-2 culture

The bacterial strain used in this study, *Priestia megaterium* NCT-2, was originally isolated from secondary salinized soil in greenhouse facilities located in Chongming District, Shanghai, China, which had a cultivation history of over 10 years. The strain was identified based on its 16S rRNA gene sequence analysis and morphological characteristics. To ensure its availability to the scientific community, this strain has been deposited in the China General Microbiological Culture Collection Center (CGMCC) under the accession number CGMCC No. 4698. *P. megaterium* NCT-2 was specifically selected for this study due to its demonstrated high tolerance to saline conditions, capable of growth in media containing up to 60 g L^−1^ NaCl (approximately 1.03 M), which significantly exceeds the salinity level (200 mM, or ~11.7 g L^−1^ NaCl) that is typically harmful to plants and challenges many microbes. This robust salt tolerance, combined with its previously observed plant growth-promoting traits, made it an ideal candidate for investigating microbial remediation of saline soils.

The *P. megaterium* NCT-2 was inoculated into 500 mL flasks containing 100 mL inorganic salt medium (KNO_3_ as a nitrogen source) and cultured at 200 r min^−1^ (rotation speed) and 35 °C for 10 h. Three fermentation media based on the key chemical components of the secondary salinized soil were developed to better understand *P. megaterium* NCT-2’s remediation mechanism and adaptability strategy to nitrate and salinity. The seed solution of the strain was inoculated into a 500 mL flask containing 100 mL of fermentation medium at 2.0% inoculum and cultured at 200 r min^−1^ and 35 °C for 72 h. The bacterial growth curve (OD_600_) was measured during the culture period using a Tecan M200 Pro microplate spectrophotometer (Tecan Austria GmbH, Salzburg, Austria) every 3 h. After culturing for 48 h, cells were harvested by centrifugation at 4 °C and 5,000 rpm for 15 min and used for transcriptomic sequencing. The composition of inorganic salt medium is as follows: KNO_3_ 1 g L^−1^, KCl 1 g L^−1^, FeSO_4_·7H_2_O 0.01 g L^−1^; MgSO_4_·7H_2_O 0.5 g L^−1^; CaCl_2_ 0.01 g L^−1^; KH_2_PO_4_ 0.5 g L^−1^; glucose 10 g L^−1^. Three media were formulated with the main chemical components of the greenhouse salinized soil. The formulations are as follows: Control group (CK): glucose 20 g L^−1^, (NH_4_)_2_SO_4_ 1.89 g L^−1^ (N content 400 mg L^−1^), KH_2_PO_4_ 1.0 g L^−1^, and MnSO_4_ 0.05 g L^−1^. Treatment 1 (NCTa): glucose 20 g L^−1^, Ca(NO_3_)_2_ 2.34 g L^−1^ (N content 400 mg L^−1^), KH_2_PO_4_ 1.0 g L^−1^, MnSO_4_ 0.05 g L^−1^. Treatment 2 (NCTb): glucose 20 g L^−1^, Ca(NO_3_)_2_ 2.34 g L^−1^ (N content 400 mg L^−1^), KH_2_PO_4_ 1.0 g L^−1^, MnSO_4_ 0.05 g L^−1^, NaC1 60 g L^−1^. LB medium formula: NaCl 10 g L^−1^, tryptone 10 g L^−1^, yeast extract 5 g L^−1^, agar powder 20 g L^−1^. All chemicals were from Sigma-Aldrich.

### 2.2 Test of salt ions in soil

Soil was collected by random sampling on September 8, 2019, from greenhouse areas (idle period, soil depth 0–20 cm) at Guangji road, Minhang district, Shanghai city in China (Shanghai city vegetable production and marketing cooperative) (121^°^33′14″E, 31^°^0′3”N). The fundamental physicochemical properties of the soil were as follows (methods described in the reference): pH (1:2.5 H₂O) 7.8 ± 0.3, initial electrical conductivity (EC) 2.35 ± 0.15 S m^−1^, and a loam texture. The soil was air-dried, crushed, and passed through a 2-mm sieve before use.

The *Priestia megaterium* NCT-2 strain used in this study was originally isolated from secondary salinized soil (Chu et al., 2018). To prepare the microbial agent, the NCT-2 strain was inoculated into a mineral salt medium with KNO₃ as the nitrogen source (medium composition in g L^−1^: KNO₃, 1; KCl, 1; FeSO₄·7H₂O, 0.01; MgSO₄·7H₂O, 0.5; CaCl₂, 0.01; KH₂PO₄, 0.5; glucose, 10; pH 7.0). The culture was incubated at 35 °C and 180 rpm for 24 h to obtain the seed culture. Subsequently, the microbial agent was prepared using the same medium as the fermentation medium and humic acid as the carrier. The viable bacterial count, determined by the plate count method, reached over 2 × 10^8^ CFU g^−1^ in the final agent.

Soil samples equivalent to 1.8 kg of oven-dried soil (accurate to 0.01 g) were pre-incubated in pots. The treatment groups were as follows: Control group one (CK): no humic acid, no application of NCT-2 agent; Control group two (HA): addition of humic acid only (in an amount equal to that contained in the NCT-2 agent) but without NCT-2; NCT-2 strain treatment group (NCT-2): addition of the complete NCT-2 agent (containing both humic acid and the bacterial strain). The humic acid control was set to exclude the potential effect of the humic acid carrier itself, thereby better illustrating the specific effect of the NCT-2 strain. Each treatment was set up with three replicates (*n* = 5).

The soil moisture content in all pots was adjusted to 60% of the water-holding capacity (WHC) using deionized water. Subsequently, all pots were incubated at 25°.

C in the dark for 30 days. During the incubation, water loss was compensated for every 2 days by adding deionized water as needed.

After 30 days of the experiment, soil samples were collected to determine soil salt ions. Soil samples were air-dried and screened at 0.15 mm. NH_4_^+^ and NO_3_^−^ were extracted with 2 M KCl at a soil/extractant ratio of 1:5 after shaking for 60 min at 250 rpm and 25 °C (Li et al., 2012). Then the extract was filtered through double loop quantitative filter paper (Whatman, China) and was analyzed on a CleverChem ONE spectrophotometer (Alliance company, France) by extraction with KCl solution - automated method with segmented flow analysis (Li et al., 2012; You et al., 2021). The contents of Cl^−^, SO₄^2−^, and HCO₃^−^ were determined by ion chromatography (ThermoFisher, Germany). The contents of Na^+^, K^+^, Ca^2+^, and Mg^2+^ were determined using inductively coupled plasma optical emission spectrometry (ThermoFisher, Germany) (Richard and Donald, 1996).

### 2.3 Culture experiments with ^15^N isotope labeling

This section aims to trace the metabolic fate of nitrogen in *P. megaterium* NCT-2 using ^15^N isotope labeling. The goal was to identify and quantify the key pathways and products of nitrogen transformation under different oxygen conditions. Given that certain *P. megaterium* species possess both assimilatory nitrate reduction pathways and the potential for dissimilatory processes (like denitrification) under oxygen limitation, experiments were conducted under both aerobic and anaerobic conditions. This comparative approach is crucial for elucidating the complete picture of nitrogen metabolism in *P. megaterium* NCT-2, as the available oxygen significantly influences the enzymatic pathways activated, leading to distinct end products (e.g., cellular biomass vs. gaseous N_2_O).

#### 2.3.1 Aerobic culture experiments

*Priestia megaterium* NCT-2 was inoculated into an inorganic salt medium with K^15^NO_3_ as a nitrogen source at 2.0% inoculum and incubated (200 r min^−1^ and 35 °C for 78 h). The sterile medium was used as a control group. At 0 h, 3 h, 6 h, 12 h, 24 h, 36 h, 60 h, and 72 h, the culture medium was centrifuged at 4 °C and 5,000 rpm for 10 min, and the supernatant was collected. Cells were collected after repeated suspension, centrifugation, and washing in phosphate buffer (30 mmol Na_2_HPO_4_ + 20 mmol K_2_HPO_4_). The contents of NO_3_^−^, NO_2_^−^, and NH_4_^+^ in the supernatant were determined by CleverChem ONE spectrophotometer (Alliance company, France). The kjeldahl nitrogen analyzer was used to determine the total nitrogen content in supernatants and cells. The dry cell weight was measured by weighing. The ^15^N atomic percent (atom%) of NO_3_^−^, NO_2_^−^, and NH_4_^+^ in supernatants and the ^15^N atom% in cells were determined by a stable isotope mass spectrometer (ThermoFisher, Germany), respectively.

#### 2.3.2 Anaerobic culture experiments

*Priestia megaterium* NCT-2 was inoculated into a 250 mL fermentation flask containing 80 mL of inorganic salt medium with K^15^NO_3_ as a nitrogen source, and cultured in an anaerobic incubator at 35 °C. At 0 h, 3 h, 6 h, 12 h, 24 h, 36 h, 60 h, and 72 h, gas samples were collected using a 25 mL closed syringe (with stopper) and injected into a vacuum bag. The N_2_O concentration was measured by a greenhouse gas analyzer, and the N_2_O-^15^N atom% was measured by a stable isotope mass spectrometer. Similarly, the seed liquid was inoculated into a medium with ^15^NH_4_NO_3_ as a nitrogen source. The content and ^15^N atom % of NO_3_^−^, NO_2_^−^, NH_4_^+^ were analyzed on a CleverChem ONE spectrophotometer (Alliance company, France) by extraction with KCl solution - automated method with segmented flow analysis (Li et al., 2012; You et al., 2021). Total nitrogen was determined by an Element analyzer (ThermoFisher, Germany).

### 2.4 Functional validation of *nasC* and *nasD* genes by gene knockout and complementation

#### 2.4.1 Rationale for gene selection and confirmation of target genes

The assimilatory nitrate reductase gene (*nasC*) and nitrite reductase gene (*nasD*) were selected for functional validation because they encode the key enzymes in the dissimilatory nitrate reduction pathway, which is central to the proposed mechanism of nitrate removal by *P. megaterium* NCT-2 from saline soil. This selection was based on our laboratory’s prior genomic sequencing of the NCT-2 strain and preliminary pathway analysis.

#### 2.4.2 Construction of knockout vectors

The knockout vectors were constructed using an allele replacement strategy via homologous recombination. The upstream and downstream homology arms for *nasC* (300 bp for *nasC*-L and 297 bp for *nasC*-R) and for *nasD* (312 bp for *nasD*-L and 309 bp for *nasD*-R) were amplified from the genomic DNA of wild-type *P. megaterium* NCT-2. A chloramphenicol resistance gene (Cm^r^) was amplified from plasmid pBR325. The fragments [Homology Arm L - Cm^r^ - Homology Arm R] for both *nasC* and *nasD* were assembled by overlap PCR, resulting in the *nasC*L-Cm-*nasC*R and *nasD*L-Cm-*nasD*R cassettes, respectively. These cassettes were then cloned into the *Bam*HI/BglII site of the *E. coli-P. megaterium* shuttle vector pHIS1525. The resulting plasmids, designated pHIS1525-nasC and pHIS1525-nasD, were verified by sequencing in *E. coli* JM109. The primer sequences and PCR conditions are listed in Supplementary Table S1.

#### 2.4.3 Generation and verification of knockout mutants

The recombinant plasmids pHIS1525-nasC and pHIS1525-nasD were independently transformed into wild-type *P. megaterium* NCT-2 protoplasts (Biedendieck et al., 2011). Transformants were initially selected on LB agar plates containing chloramphenicol.

To ensure the selection of true double-crossover mutants and to cure the replicative pHIS1525 plasmid, a critical screening step was implemented. Positive transformants were subcultured for more than 10 generations in antibiotic-free LB medium to allow for plasmid loss. These cultures were then plated onto LB agar without antibiotics. Individual colonies were replica-plated onto LB agar with and without chloramphenicol. Colonies that grew on the non-selective medium but failed to grow on the chloramphenicol-containing medium (i.e., chloramphenicol-sensitive) were selected as potential double-crossover mutants.

Genomic DNA from these potential mutants was subjected to a rigorous PCR-based verification. Using primers that anneal to regions flanking the upstream and downstream homology arms, a PCR product of the expected size (*nasC*L-Cm-*nasC*R: 1257 bp; *nasD*L-Cm-*nasD*R: 1281 bp) was obtained. The identity of these PCR products was conclusively confirmed by Sanger sequencing, verifying the precise replacement of the wild-type allele with the knockout cassette. To definitively rule out single-crossover events (plasmid integration) or the persistence of the free plasmid, PCR was performed using primers specific to the pHIS1525 vector backbone sequence outside the cloned region. Clones that yielded a negative result in this PCR were considered clean, unambiguous chromosomal knockouts. The two verified mutant strains were designated *P. megaterium* NCT-2-Δ*nasC* and NCT-2-Δ*nasD*.

#### 2.4.4 Construction of complementation strains and phenotypic assay

Complementation strains were constructed to verify that the observed phenotypes were due to the specific gene knockouts. The full-length *nasC* or *nasD* gene, including its native promoter, was amplified and fused to a kanamycin resistance gene (Km) via overlap PCR. The fusion fragments (*nasC*-Km and *nasD*-Km) were cloned into the *P. megaterium* expression vector pWH1520. The resulting plasmids, pWH1520-nasC-Km and pWH1520-nasD-Km, were transformed into the corresponding mutant strains to generate the complementation strains NCT-2-nasC-Km and NCT-2-nasD-Km.

The wild-type, knockout mutants, and complementation strains were inoculated into an inorganic salt medium with nitrate as the sole nitrogen source. Bacterial growth (OD_600_) was monitored, and the concentrations of NO_3_^−^, NO_2_^−^, and NH_4_^+^ in the supernatant were determined to assess the functional impact of gene knockout and complementation (Li et al., 2012; You et al., 2021a).

### 2.5 Transcriptome sequencing (RNA-seq)

Transcriptome sequencing was performed on *P. megaterium* NCT-2 cells grown under different salt concentrations (as described in section 2.1, *P. megaterium* NCT-2 culture) to investigate the effects of salt stress on gene expression and metabolic mechanisms. Samples were collected during the mid-log phase of growth for RNA extraction, with each treatment condition including three biological replicates. Prior to RNA isolation, cells were harvested by centrifugation at 4 °C and 10,000 × g for 10 min, and the pellet was washed twice with phosphate-buffered saline (PBS) to remove residual media components.

#### 2.5.1 RNA extraction

Total RNA of *P. megaterium* NCT-2 was extracted using TRIzol® reagent according to the manufacturer’s instructions (Invitrogen, USA), and genomic DNA was removed using DNase I (Takara, China). RNA quality was determined with an Agilent 2,100 Bioanalyzer, and RNA was quantified using an ND-2000 (NanoDrop Technologies). High-quality RNA samples (OD260/280 = 1.8 ~ 2.0, OD260/230 ≥ 2.0, RNA Integrity Number (RIN) ≥ 6.5, 23S:16S ≥ 1.0, total amount≥100 ng μL^−1^, concentration≥2 μg) were used for library construction and Real-time PCR.

#### 2.5.2 Library construction and sequencing

RNA libraries were constructed using the TruSeqTM RNA sample preparation Kit from Illumina (San Diego, CA). The rRNA was removed using the Ribo-Zero Magnetic kit (epicenter), and the mRNA was randomly fragmented into small fragments of about 200 bp. Double-stranded cDNA was synthesized by reverse transcription using mRNA template, random primers, and SuperScript double-stranded cDNA synthesis kit (Invitrogen, CA). The second strand of cDNA was synthesized by dUTP instead of dTTP. Double-stranded cDNA was blunt-ended by adding End Repair Mix. Then the 5′ end was phosphorylated, an ‘A’ base is added to the 3′ end, and it is ligated into a Y-shaped sequencing adapter. The second strand of cDNA containing dUTP was eliminated with UNG enzyme, so that only the first strand of cDNA was included in the library.

The enriched library was extracted by PCR amplification with Phusion DNA polymerase (NEB) for 15 cycles. Quantification was performed with TBS380 (Picogreen), and RNA-seq paired-end sequencing was performed using Illumina HiSeq X Ten (2 × 150 bp). Subsequently, the sequencing results were compared, annotated, and analyzed.

#### 2.5.3 Bioinformatics analysis

The data generated from the Illumina platform were used for bioinformatics analysis. All of the analyses were performed using the free online platform of Majorbio Cloud Platform^1^ from Shanghai Majorbio Bio-pharm Technology Co., Ltd. The major software and parameters are as follows. High-quality reads in each sample were mapped to the reference genome of *Priestia megaterium* NCT-2 (assembly ASM33487v3, obtained from NCBI RefSeq) using Bowtie2.^2^ Analysis tool: Bowtie2 (see footnote 2).

#### 2.5.4 rRNA contamination assessment

In this step, randomly selected 10,000 raw reads in each sample are aligned to the Rfam database^4^ with the blast method. Based on the annotation results, the percentage of rRNA in each sample is calculated, which is estimated as rRNA contamination. The rRNA contamination was less than 5% in all samples in Supplementary Table S9. Analysis tool: Blast.

#### 2.5.5 Expression analysis

Gene and isoform abundances were quantified using RSEM (v1.3.0) (Li and Dewey, 2011). RSEM employs an Expectation–Maximization (EM) algorithm to compute maximum likelihood abundance estimates, accounting for paired-end reads, fragment length distributions, and sequencing quality scores. Expression levels were reported in both FPKM (Fragments Per Kilobase of transcript per Million mapped reads) and TPM (Transcripts Per Million) units. These normalized metrics eliminate the confounding effects of gene length and sequencing depth variations, thereby enabling direct comparison of gene expression levels across different samples (Wagner et al., 2012). For downstream differential expression analysis, we utilized the TPM values due to their superior cross-sample comparability.

Raw read counts for each gene were obtained from the alignment files. Differential expression analysis was performed using the DESeq2.^5^ Genes with an adjusted *p*-value (Benjamini-Hochberg procedure) of less than 0.05 and an absolute fold change greater than 2 (|log2FoldChange| > 1) were considered statistically significant and differentially expressed.

The Gene Ontology^6^ project provides an ontology of defined terms representing gene properties, which covers three domains: Cellular Component, Molecular Function, and Biological Process. GO enrichment analysis will find gene ontology (GO) terms in which differentially expressed genes (DEGs) are enriched. It also helps to illustrate the difference between two particular samples on functional levels.

Goatools^7^ is used to identify statistically significantly enriched GO terms using Fisher’s exact test. The purpose of performing false discovery rate (FDR) Bonferroni correction is to reduce the Type-1 error by bonferroni, holm-bonferroni method (Holm), Benjamini-Yekutieli procedure (BY), Benjamini-Hochberg (BH) (multiple hypothesis test method). After multiple testing corrections, GO terms with adjusted *p*-value ≤ 0.05 are significantly enriched in DEGs.

Different expressed genes (DEGs) usually interact with each other *in vivo* to play roles in certain biological functions. Compared with the whole genome background, Kyoto Encyclopedia of Genes and Genomes enrichment analysis could identify the most important biological metabolic pathways and signal transduction pathways of DEGs.

KOBAS 2.0^8^ is used to identify statistically significantly enriched pathways using Fisher’s exact test. The purpose of performing FDR correction is to reduce the Type-1 error by bonferroni, Holm, BY, BH (multiple hypothesis test method). The calculating formula of the p-value and corrected p-value is similar to that in GO analysis. After multiple testing corrections, we chose pathways with a p-value ≤ 0.05, which are significantly enriched in DEGs.

### 2.6 Real-time PCR (RT-PCR)

Real-time PCR was performed using TB Green® Premix Ex Taq™ II (Takara, China) according to the commercial instructions. The relative expression levels of genes were calculated by the 2^-△△Ct^ method (Gutsch et al., 2019). The primers used in this experiment are shown in Supplementary Table S3 for details.

### 2.7 Data analysis

All experiments were performed with three independent biological replicates (n = 3). Data are presented as the mean ± standard deviation (SD). Prior to statistical analysis, the normality of data distribution was verified using the Shapiro–Wilk test, and homogeneity of variances was confirmed using Levene’s test. One-way analysis of variance (ANOVA) was used to determine significant differences between all experimental treatments, followed by Tukey’s post-hoc test for multiple comparisons. The levels of significance are denoted as *p* < 0.05, *p < 0.01, and **p < 0.001. All graphs were generated using Origin 9.0 software and the Genescloud platform.^9^ Statistical analysis was performed using IBM SPSS Statistics software (version 22.0).

## 3 Results

### 3.1 Salt ion content in soil

**Figure 1:**
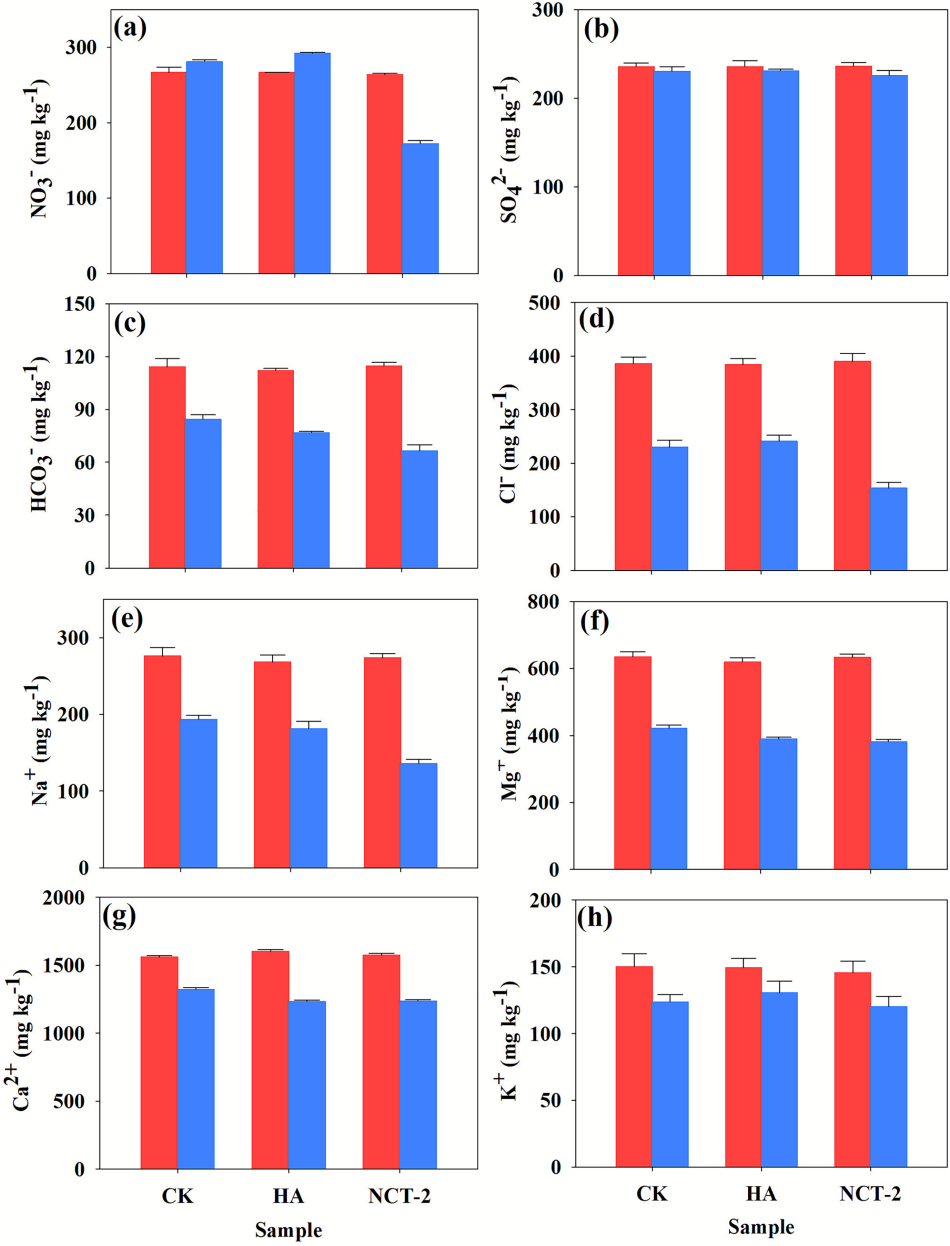
The content experiment initial and final salt ions in all soil samples. The red column represented the content of salt ions in the soil on the 0 d of the experiment, and the blue column represented the salt ion content in the soil on the 30 d of the experiment. The abscissa represented the sample name. Error bars represent the standard deviation from three independent biological replicates (*n* = 3). **(a)** Nitrate, **(b)** Sulfate, **(c)** Bicarbonate, **(d)** Chloride, **(e)** Sodium ion, **(f)** Magnesium ion, **(g)** Calcium ion, **(h)** Potassium ion.

In order to understand the prospect of the NCT-2 agent in improving secondary salinized soil, the effect of this agent on soil salt ions was analyzed in this experiment. The result found that application of NCT-2 agent significantly decreased the contents of NO_3_^−^, Na^+^, Cl^−^, and HCO_3_^−^ in the soil compared with the two control groups and the 0 d test (*p* < 0.05) (Figure 1). Compared with the control (CK), NO_3_^−^ decreased by 41.67%, Cl^−^ decreased by 33.34%, Na^+^ decreased by 29.98%, and HCO_3_^−^ decreased by 27.33% (*p* < 0.05) (Figure 1). Compared with the humic acid treatment (HA), NO_3_^−^ decreased by 46.02%, Cl^−^ decreased by 36.44%, Na^+^ decreased by 25.34% and HCO_3_^−^ decreased by 13.60% (*p* < 0.05) (Figure 1). It can be seen that the removal effect of NO_3_^−^ was the best, followed by Cl^−^ and Na^+^. In all samples, the contents of K^+^, Ca^2+^, Mg^2+^, and SO_4_^2−^ had no significant changes, indicating that the application of NCT-2 agent had no effect on them (Figure 1). These results indicated that *P. megaterium* NCT-2 agent has a good application prospect in the improvement of secondary salinized soil.

### 3.2 Nitrate metabolic pathway of *Priestia megaterium* NCT-2

#### 3.2.1 Growth curves of strains in different salt environments

**Figure 2:**
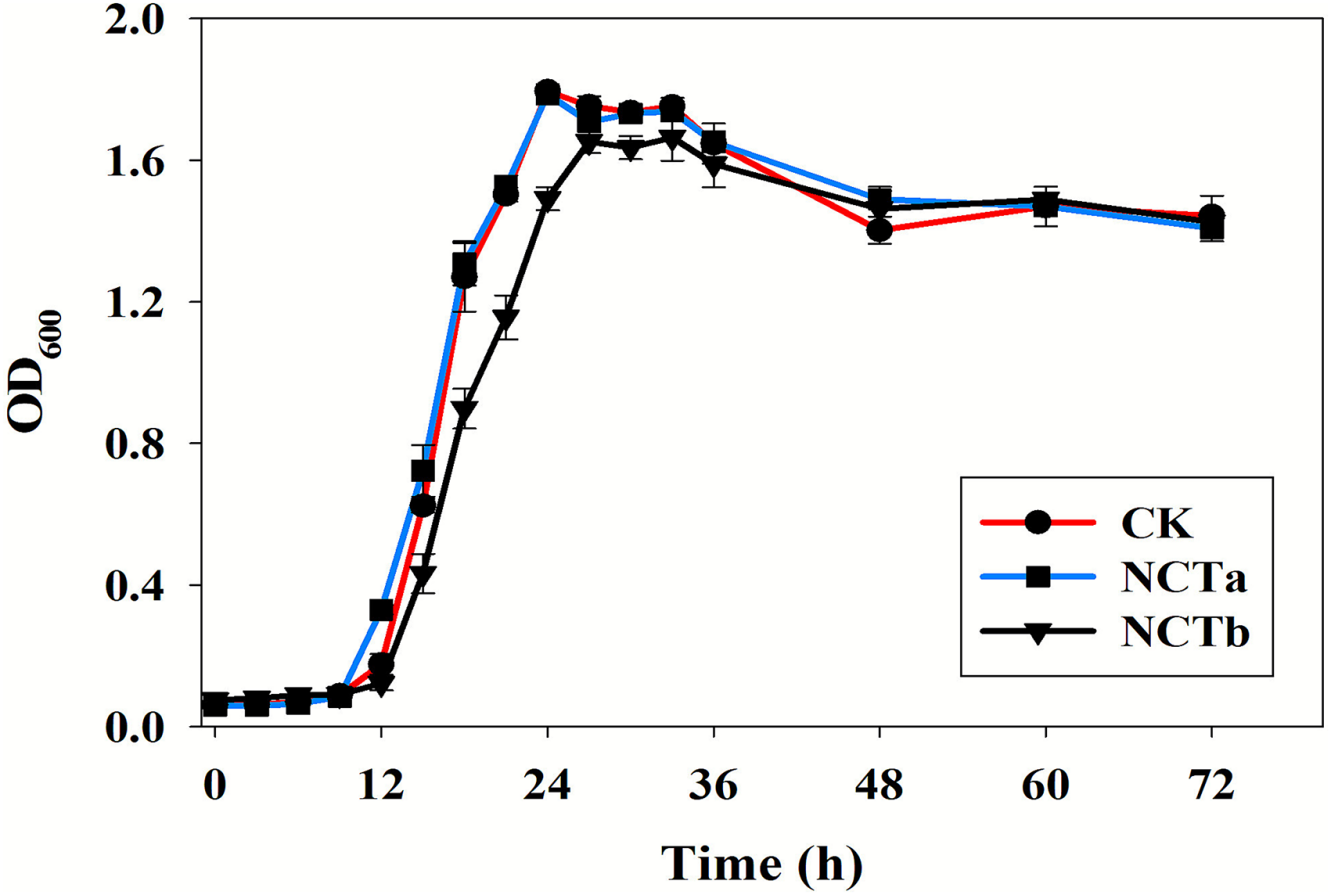
Growth curve of *P. megaterium* NCT-2 in the medium (CK) with ammonium as a nitrogen source, the medium with nitrate as a nitrogen source (NCTa), and the medium with nitrate and salt stress (NCTb). Error bars represent the standard deviation from three independent biological replicates (*n* = 3).

The previous experimental results found that the most salt ions removed by NCT-2 inoculant were NO_3_^−^, followed by Cl^−^ and Na^+^. Therefore, we further analyzed the tolerance of this strain to NO_3_^−^, Cl^−^, and Na^+^ under pure culture conditions. The strain was inoculated into the medium (CK) with ammonium as a nitrogen source, the medium with nitrate as a nitrogen source (NCTa), and the medium with nitrate and salt stress (NCTb). The results demonstrated that the growth of *P. megaterium* NCT-2 was similar in ammonium (CK) and nitrate (NCTa) as nitrogen sources (Figure 2). These findings confirmed that the strain can transform and utilize nitrate. In addition, the strain growth was slower than NCTa from 0–48 h in salt stress (NCTb) (Figure 2). However, the final growth was the same for the three treatments (Figure 2). Therefore, the results of this analysis show that *P. megaterium* NCT-2 is capable of efficiently using nitrate in salt stress conditions.

#### 3.2.2 Reduction dynamics of NO_3_^−^

**Figure 3:**
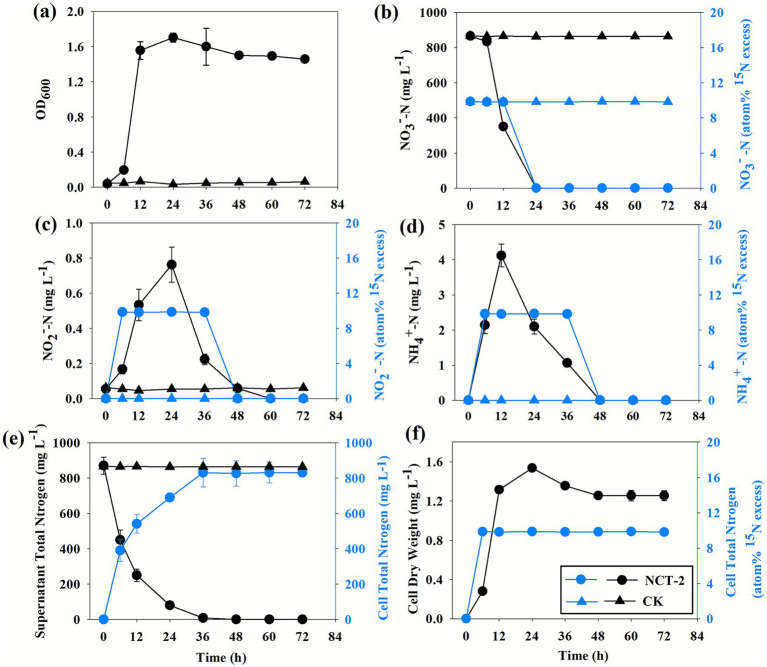
*Priestia megaterium* NCT-2 was cultured under aerobic conditions. **(a)** Growth curve, **(b)** NO_3_^−^ content and NO_3_^−^-^15^N atom% in the medium, **(c)** NO_2_^−^ content and NO_2_^−^-^15^N atom% in the medium, **(d)** NH_4_^+^ content and NH_4_^+^-^15^N atom% in the medium, **(e)** the total nitrogen of medium and the total nitrogen of cells, **(f)** the cell dry weight and cells ^15^N atom %. Error bars represent the standard deviation from three independent biological replicates (*n* = 3).

Even though it has been demonstrated that *P. megaterium* NCT-2 can transform and utilize nitrate, the nitrate metabolic pathway of *P. megaterium* NCT-2 remains unknown. Therefore, this study explored the reduction dynamics of NO_3_^−^ by *P. megaterium* NCT-2. The growth curve, nitrogenous compounds, and ^15^N atom% were determined in NO_3_^−^ as a nitrogen source. These results showed that the logarithmic growth phase of the strain was comprised of 6–18 h (Figures 3a,b). After 18 h of culture, NO_3_^−^ had been entirely metabolized, and approximately 0.23 mg L^−1^ NO_2_^−^ and 2.2 mg L^−1^ NH_4_^+^ had been accumulated in the medium, respectively. Subsequently, the NO_2_^−^ and NH_4_^+^ were gradually decreased and fully utilized (Figures 3b–d). The NO_3_^−^ content of supernatant was 0 mg L^−1^ at 18 ~ 72 h, indicating that no NO_3_^−^ was generated. Furthermore, NO_2_^−^-^15^N and NH_4_^+^-^15^N atom% were identical with NO_3_^−^-^15^N atom% of the marker during the culture period, which further proved that the production of NO_2_^−^ and NH_4_^+^ was derived from NO_3_^−^. Therefore, the reduction process of NO_3_^−^ was NO_3_^−^—NO_2_^−^—NH_4_^+^.

The changes in cell dry weight and cell total nitrogen were similar to the growth curve. The cell dry weight and cell total nitrogen gradually increased in the logarithmic growth phase, and then stabilized in the stationary phase (Figures 3e,f). On the contrary, the total nitrogen content first gradually decreased and then stabilized in the supernatant (Figures 3e,f). These indicated that *P. megaterium* NCT-2 could transport NO_3_^−^ into cells. Thus, *P. megaterium* NCT-2 primarily utilizes the aerobic assimilation pathway to metabolize nitrate.

#### 3.2.3 Dissimilatory nitrate rduction to ammonium process of *Priestia megaterium* NCT-2

**Figure 4:**
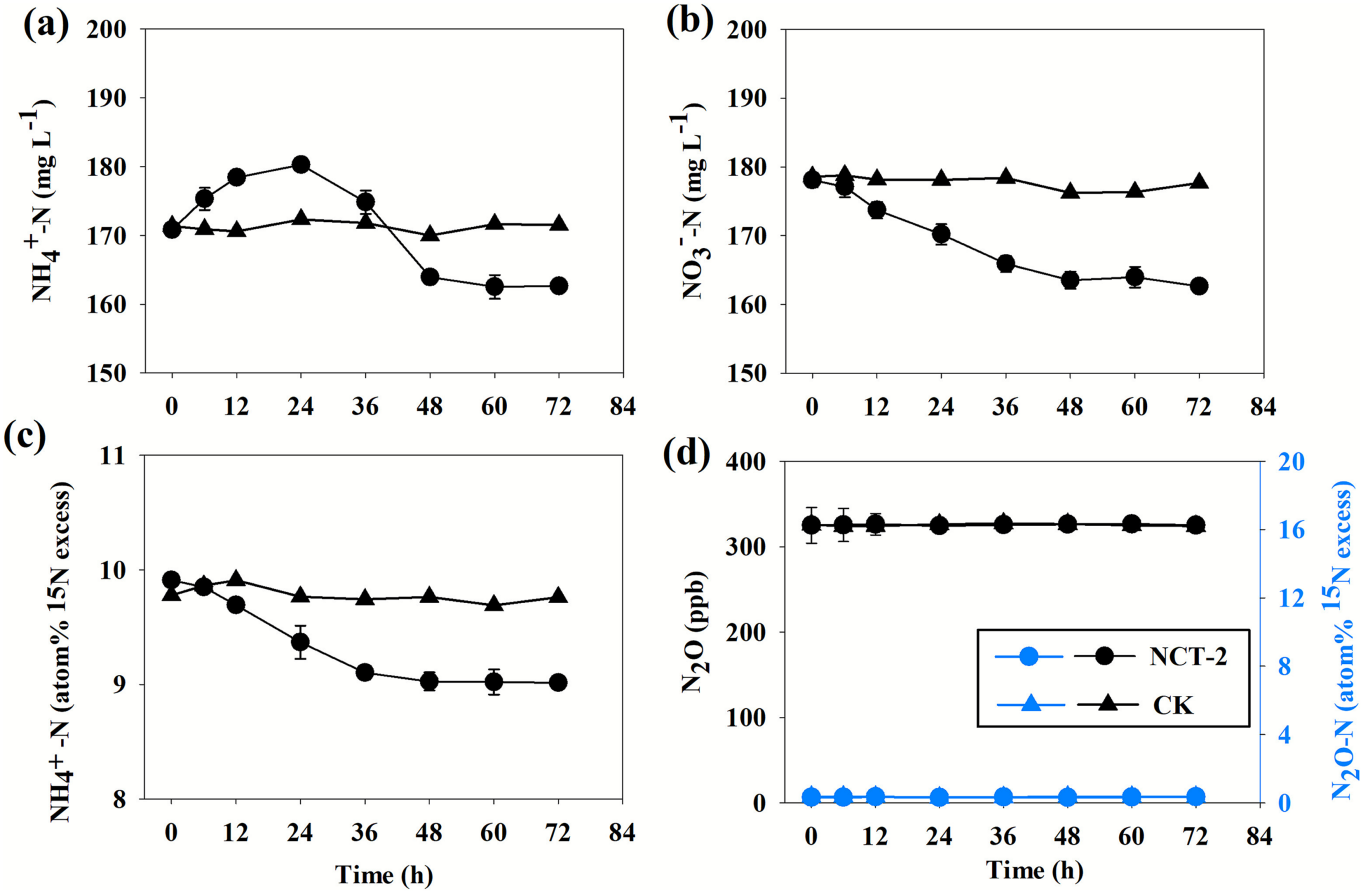
*Priestia megaterium* NCT-2 was cultured under anaerobic conditions. In the medium, **(a)** NH_4_^+^ content, **(b)** NO_3_^−^ content, **(c)** NH_4_^+^-^15^N atom%, **(d)** N_2_O content and N_2_O-^15^N atom%. Error bars represent the standard deviation from three independent biological replicates (*n* = 3).

When ^15^NH_4_^+^ and NO_3_^−^ are present in a medium, the N dilution occurs through DNRA or cell N mineralization. In order to investigate the DNRA process, *P. megaterium* NCT-2 (NCT-2) was cultured in medium containing ^15^NH_4_NO_3_ as a nitrogen source, and sterile medium (CK) serving as a control, in anaerobic conditions. The NH_4_^+^ in the medium first gradually increased and then decreased (Figure 4a). Over the course of the culture period, the NO_3_^−^ level decreased, whereas the metabolized NO_3_^−^ quantity was only 16 mg L^−1^ (Figure 4b). These results suggested that the strain consumed NO_3_^−^ and produced NH_4_^+^. The initial NH_4_^+^-^15^N atom% in the medium was 9.98%, which was decreased to 9.69% after 12 h and 9.46% after 24 h (Figure 4c). This demonstrated that NH_4_^+^-^15^N was continuously diluted by unlabeled NH_4_^+^. NO_3_^−^ was the only unlabeled nitrogen source, so the unlabeled NH_4_^+^ can only come from NO_3_^−^. These results indicated that the strain could carry out the DNRA pathway, even though its NO_3_^−^ metabolism was significantly lower than that of the assimilation pathway.

#### 3.2.4 Denitrification

Some microorganisms can use NO_3_^−^ to produce N_2_O and N_2_ in anaerobic conditions. However, the finding showed that the N_2_O content was consistent with that in the air, and ^15^N atom% did not change during the culture period (Figure 4d). Thus, proving that the NCT-2 strain could not produce N_2_O and could not perform denitrification.

### 3.3 Identification of nitrate metabolic pathway in *Priestia megaterium* NCT-2 by gene knockout

The NO_3_^−^ metabolic pathway of *P. megaterium* NCT-2 may be mainly assimilation pathway. Therefore, the key enzyme genes (nitrate reductase gene *nasC* and nitrite reductase gene *nasD*) in the assimilation pathway were further knocked out to verify the NO_3_^−^ metabolic pathway.

**Figure 5:**
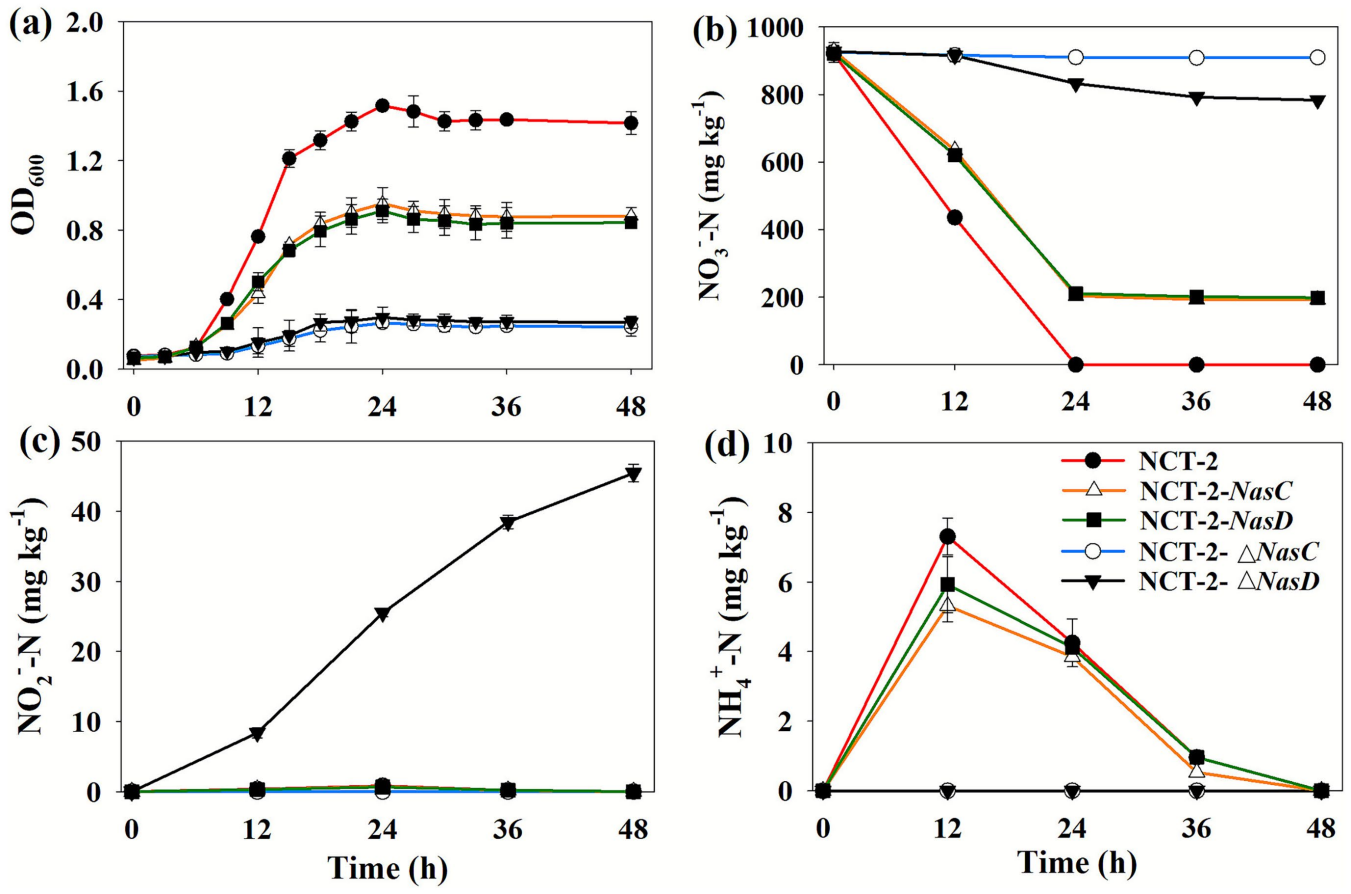
**(a)** Growth curve of *P. megaterium* NCT-2, *P. megaterium* NCT-2-△*nasC* mutant, *P. megaterium* NCT-2-△*nasD* mutant, complement strain *P. megaterium* NCT-2-*nasC*-Km, and complement strain *P. megaterium* NCT-2-*nasD*-Km. **(b)** NO_3_^−^, **(c)** NO_2_^-,^ and **(d)** NH_4_^+^ contents in the medium. Error bars represent the standard deviation from three independent biological replicates (*n* = 3).

These construction results of the recombinant vectors, mutant strains, and complement strains of the *nasC* and *nasD* genes were shown in the Supplementary Figures S1–S3 and Results 3.1 ~ 3.3. To verify the functions of the *nasC* and *nasD* genes, the wild strain *P. megaterium* NCT-2, two mutant strains (*P. megaterium* NCT-2-△*nasC* and *P. megaterium* NCT-2-△*nasD*), and two complement strains (*P. megaterium* NCT-2-*nasC*-Km and *P. megaterium* NCT-2-*nasD*-Km) were cultured in NO_3_^−^ as a nitrogen source. The results showed that the wild strain could grow normally, and the maximum OD_600_ was observed to be 1.6 (Figure 5a). The growth of the two complement strains was about 56% of the wild strain, and the maximum OD_600_ was about 0.9 (Figure 5a). The growth of the two mutant strains was 15.63% of the wild strain, and the OD_600_ (maximum value, 0.25) was obviously lower than the wild strain and complement strains (Figure 5a). These results demonstrated that *nasC* and *nasD* genes are key genes for nitrate utilization in *P. megaterium* NCT-2.

The NO_3_^−^ was completely utilized by *P. megaterium* NCT-2 (Figure 5b). The transformation amount of NO_3_^−^ was about 80% by two complement strains (Figure 5b). The *P. megaterium* NCT-2-△*nasD* mutant transformed 14.89% of NO_3_^−^, while the *P. megaterium* NCT-2-△*nasC* mutant only used 2.18% of NO_3_^−^ (Figure 5b). Furthermore, the most NO_2_^−^ (45 mg kg^−1^) was accumulated in the medium of *P. megaterium* NCT-2-Δ*nasD* mutant (Figure 5c). During the experiment, no NO_2_^−^ accumulation was observed in the medium of *P. megaterium* NCT-2-△*nasC* mutant (Figure 5c). A small amount of NO_2_^−^ was detected in the medium of *P. megaterium* NCT-2 and two complement strains, which was then fully utilized by these strains (Figure 5c). Similarly, the NH_4_^+^ was accumulated and was subsequently utilized by *P. megaterium* NCT-2 and two complement strains, while NH_4_^+^ was not detected in the medium of two mutant strains (Figure 5d). These results suggested that the *nasC* gene is the key gene for nitrate transformation in *P. megaterium* NCT-2. The *nasD* gene is the key gene for nitrite transformation. Furthermore, transcriptomics of nitrate metabolism demonstrated that NCT-2 metabolized nitrate into glutamate metabolism (Supplementary Figure S4 and Result 3.4). These results further revealed that the assimilation pathway is the main pathway of NO_3_^−^ metabolism in *P. megaterium* NCT-2.

### 3.4 The adaptation strategies of *Priestia megaterium* NCT-2 to salt stress

Previous studies have shown that *P. megaterium* NCT-2 can metabolize nitrate in salt stress. Herein, transcriptomic analysis was used to uncover the mechanism and adaptation strategy of this strain to nitrate (CK and NCTa) and salt stress (NCTa and NCTb). The main difference between CK and NCTa samples was the nitrogen source, and the main difference between NCTa and NCTb samples was salt stress.

#### 3.4.1 Screening of significantly differentially expressed genes (DEGs)

**Figure 6:**
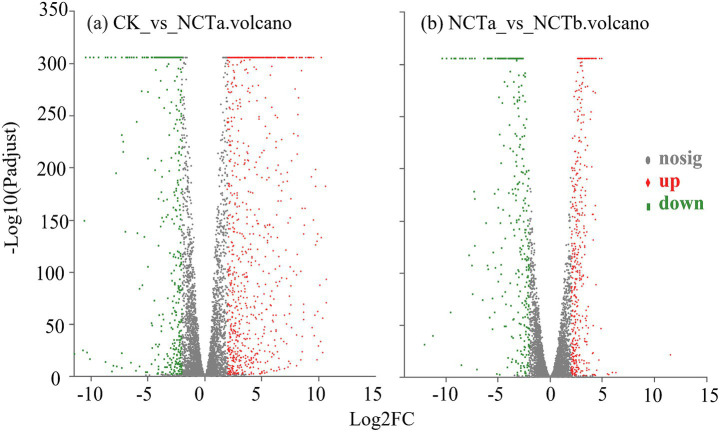
Volcano map of significantly differentially expressed genes: **(a)** CK vs. NCTa group; **(b)** NCTa vs. NCTb group. Red and green represent significantly upregulated genes and significantly downregulated genes, respectively. Each dot represents a gene.

The sequence alignment of transcriptomics is presented in Supplementary Table S4. The DEGs of *P. megaterium* NCT-2 in nitrate and salt stress were further screened according to the standard of difference significance. The FC > 4 and FDR < 0.05 are significantly up-regulated genes. The FC < 0.25 and FDR < 0.05 are significantly down-regulated genes. The result showed that there were 1,315 DEGs in the CK vs. NCTa group, including 944 significantly up-regulated genes and 371 significantly down-regulated genes (*p* < 0.05) (Figure 6a). There were 767 DEGs in NCTa vs. NCTb group, of which 448 were significantly up-regulated and 319 were significantly down-regulated (*p* < 0.05) (Figure 6b).

**Figure 7:**
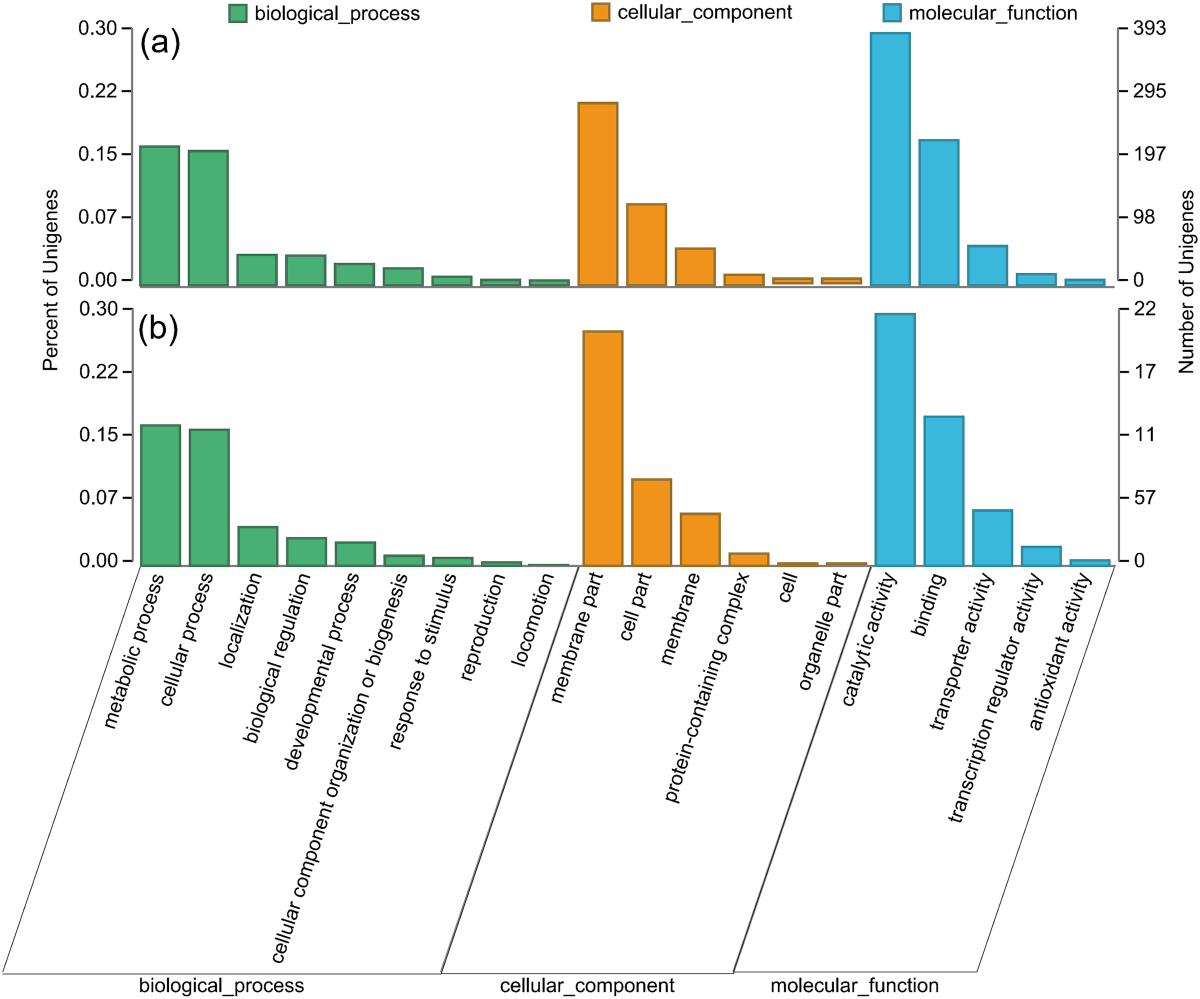
GO annotation of significantly different genes: **(a)** CK vs. NCTa group, **(b)** NCTa vs. NCTb group. The abscissa was represented the secondary classification term. The left ordinate was represented the percentage of secondary classified genes to the total number of genes. The right ordinate was represented the genes number.

**Figure 8:**
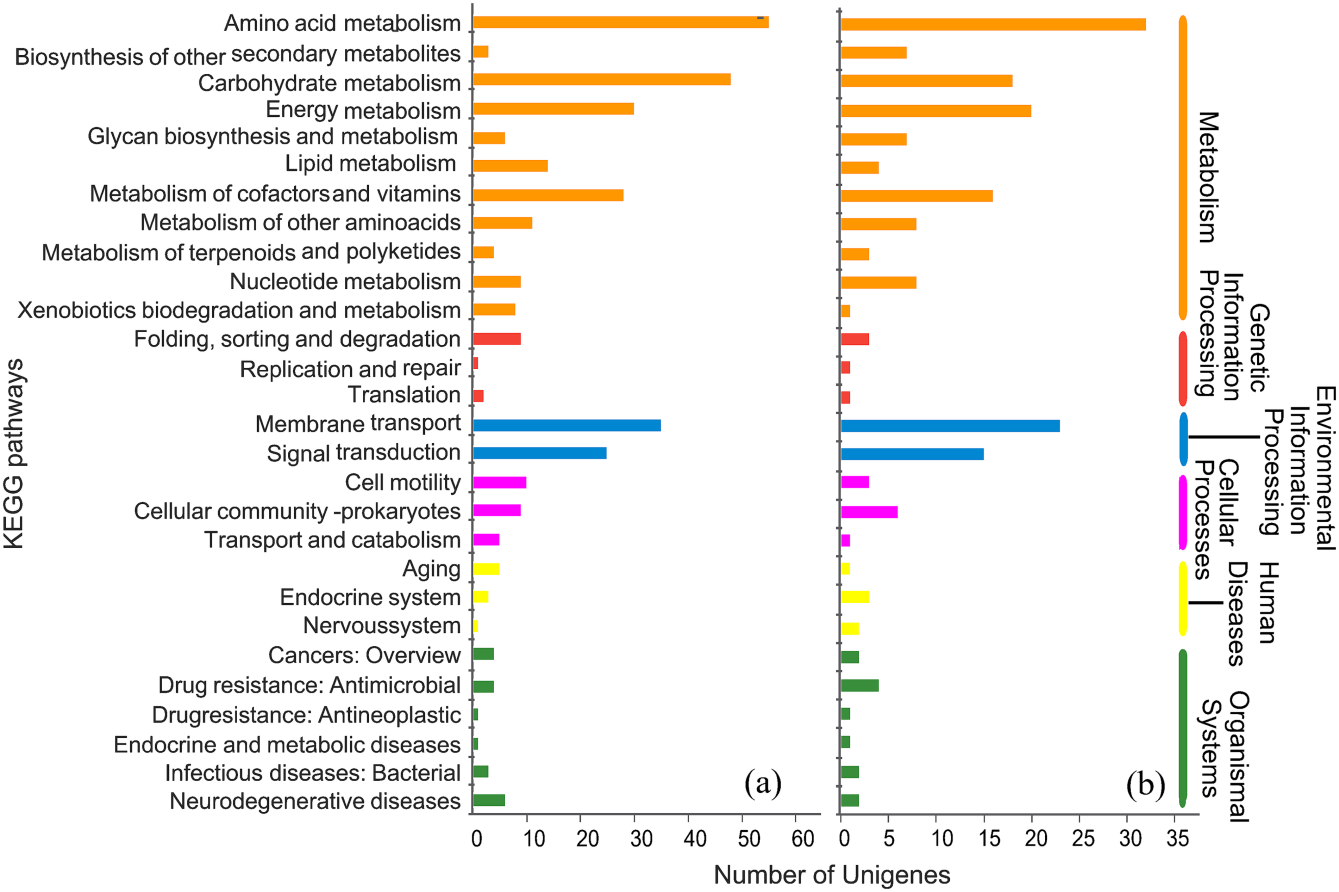
KEGG annotations of significantly different genes. **(a)** CK vs. NCTa group; **(b)** NCTa vs. NCTb group. The left ordinate was the name of the KEGG pathways. The right ordinate was the major categories of KEGG pathways. The abscissa was the number of genes.

In order to further understand the biological functions of these DEGs, GO and KEGG annotations were performed. Both groups were enriched to 26 GO secondary classification functions (Figure 7). Moreover, DEGs were mainly annotated in amino acid metabolism, carbohydrate metabolism, energy metabolism, metabolism of cofactors and vitamins, membrane transport, and signal transduction (Figure 8).

#### 3.4.2 The mechanisms of *Priestia megaterium* NCT-2 in response to salt stress

**Figure 9:**
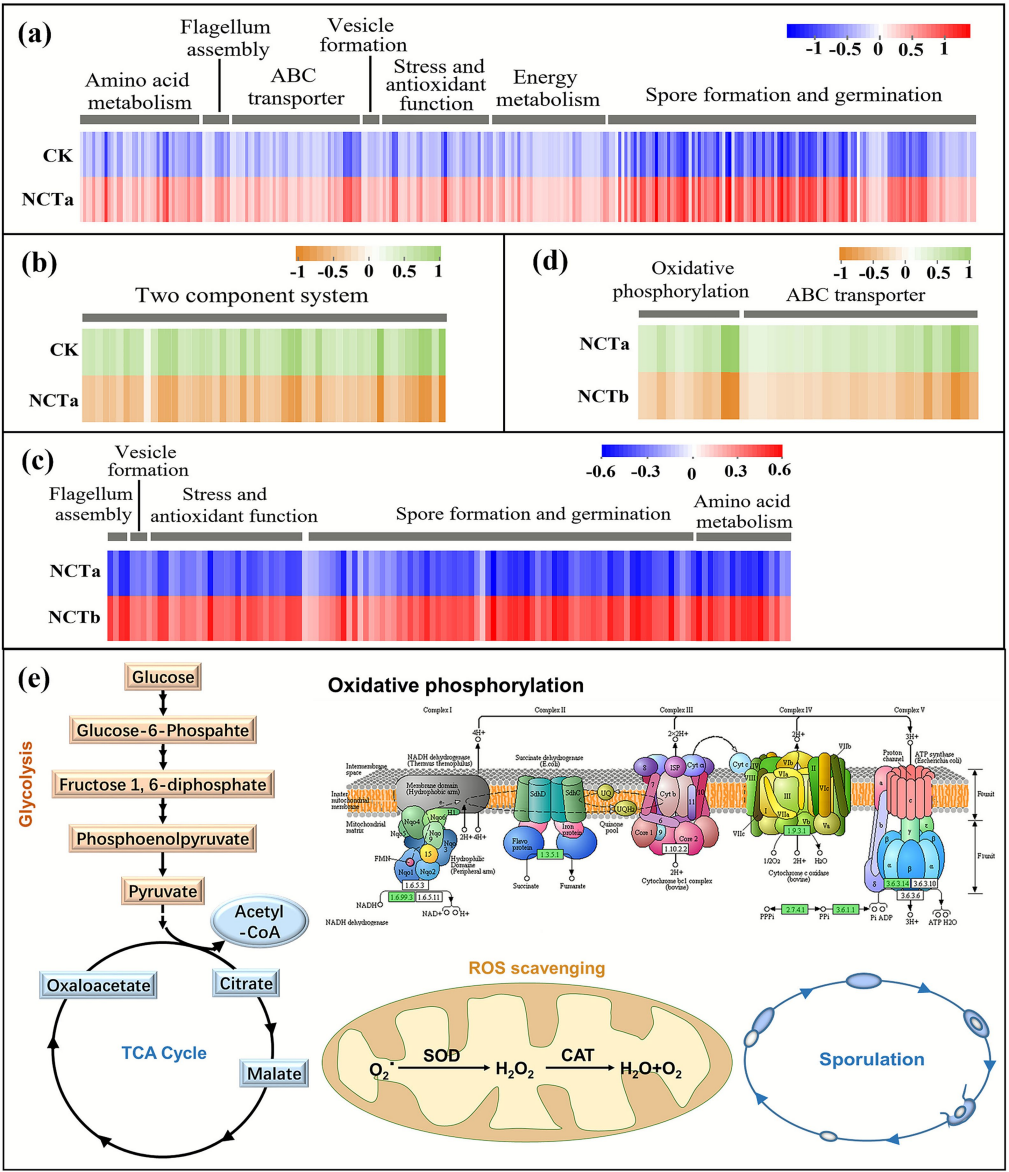
Classification of significantly different genes according to gene function. **(a)** Up-regulation gene in CK vs. NCTa group, **(b)** down-regulation gene in CK vs. NCTa group, **(c)** up-regulation gene in NCTa vs. NCTb group, **(d)** down-regulation gene in NCTa vs. NCTb group, **(e)** schematic representation of partial resistance mechanisms of *P. megaterium* NCT-2 to nitrate and salt stress, including glycolytic, tricarboxylic acid cycle, oxidative phosphorylation, ROS scavenging, and sporulation. Each column represents a gene. The data of the heatmap is the gene expression level after normalization, log (TPM + 1) and Z-score normalization (observed value-mean/standard deviation). The two arrows between the two substances indicated multi-step reaction.

We explored the functions of DEGs in more depth on the basis of GO and KEGG annotations. The results showed that DEGs were mainly related to stress regulation functions, energy metabolism, and transport processes. Specifically, in the CK vs. NCTa group, the number of up-regulated DEGs in spore formation and germination was the largest (122) (*p* < 0.05) (Figure 9a). Fifty-three DEGs were involved in amino acid metabolism (*p* < 0.05) (Figure 9a). Forty-three up-regulated DEGs were attributed to the ABC transport (*p* < 0.05) (Figure 9a). There were 41 DEGs related to energy metabolism, including oxidative phosphorylation, glycolysis, pentose phosphate pathway, and tricarboxylic acid (TCA) cycle (*p* < 0.05) (Figure 9a). The key enzymes and rate-limiting enzymes of these pathways were significantly up-regulated, including cytochrome panthenol oxidase, cytochrome C oxidase, phosphofructokinase-1, phosphoenolpyruvate carboxykinase, 6-phosphoglucose dehydrogenase, and citrate synthase. There were 36 up-regulated DEGs related to stress and antioxidant function, including universal stress protein, catalase (CAT), superoxide dismutase (SOD), Hsp20/alpha crystallin family protein, and poly-*γ*-glutamate synthase genes (*p* < 0.05) (Figure 9a). There were 10 up-regulated DEGs related to flagellar assembly, including flagellar matrix rod protein, flagellar matrix M-loop protein, and flagellar hook matrix complex protein (*p* < 0.05) (Figure 9a). Six up-regulated DEGs were functional genes in vesicle formation, including *GvpA*, *GvpL,* and *GvpF* genes (*p* < 0.05) (Figure 9a). However, the functions of down-regulated DEGs were mainly attributed two-component system (54) (*p* < 0.05) (Figure 9b).

In the NCTa vs. NCTb group, the function of up-regulated DEGs was similar to CK vs. NCTa group. There were 73 DEGs involved in spore formation and germination (Figure 9c). There were 29 up-regulated DEGs associated with stress and antioxidant functions, including universal stress protein, CAT, SOD, TetR/AcrR family transcriptional regulator (TetRs), and SOS response-associated peptidase (*p* < 0.05) (Figure 9c). There are 17 DEGs belonging to amino acid metabolism (*p* < 0.05) (Figure 9c). There were 4 up-regulated DEGs belonging to flagellum assembly (*p* < 0.05) (Figure 9c). Four up-regulated DEGs were functional genes in vesicle formation (*p* < 0.05) (Figure 9c). Down-regulated DEGs were mainly involved in ABC transport (27) and oxidative phosphorylation (11) (*p* < 0.05) (Figure 9d). Partial resistance mechanisms of *P. megaterium* NCT-2 to nitrate and salt stress are depicted in Figure 9e. In addition, all genes and expression levels are detailed in Supplementary Tables S5–S8.

In the CK vs. NCTa group, the most up-regulated genes were mainly focused on spore formation and germination, including three YjcZ family sporulation proteins (982, 846, and 787 times, respectively), outer spore coat protein *CotE* (475 times), small acid-soluble spore protein *SspI* (407 times), alpha/beta-type small acid-soluble spore protein (384 times), and spore germination protein *GerE* (382 times) (Supplementary Table S5). In NCTa compared to NCTb, the largest up-regulations were observed in amino acid metabolism, including two carbamoyl phosphate synthases (21 and 17 times), pyrroline-5-carboxylate reductase (20 times), and carbamoyl phosphate synthase small subunit (18 times). There are also two flagellins (17 and 16 times) and a sporulation protein (17 times) (Supplementary Table S7).

## 4 Discussion

Soil salinity is a serious abiotic stress, and the presence of a variety of salt ions is more serious to biological hazards (Xu et al., 2020). This study found that the NCT-2 agent could remove a large amount of NO_3_^−^, Cl^−^, and Na^+^ in soil. This proved that the agent can improve the salinity in the secondary salinized soil. Combined with the previous analysis of metabolic pathway genes (Wang et al., 2020), we speculated that this agent may have the ability to metabolize NO_3_^−^ and be resistant to NO_3_^−^, Cl^−^, and Na^+^. The pure culture experiment of the NCT-2 strain found that the strain can efficiently convert NO_3_^−^ and can grow in the environment of high NO_3_^−^, Cl^−^, and Na^+^, which verified our hypothesis. Therefore, we further analyzed the mechanisms of resistance to NO_3_^−^, Cl^−^, and Na^+^.

### 4.1 The pathway of nitrate metabolism by *Priestia megaterium* NCT-2

In aerobic conditions, NH_4_^+^ and NO_2_^−^ were generated by *P. megaterium* NCT-2. Meanwhile, NO_2_^−^-^15^N and NH_4_^+^-^15^N atom% was consistent with NO_3_^−^-^15^N atom% of the marker. Thus, NO_2_^−^ and NH_4_^+^ can came from NO_3_^−^. Enzymes of assimilation of nitrate are localized in the cytoplasm. Furthermore, the synthesis of nitrogenous matter by microorganisms also occurs in cells (Moreno-Vivián et al., 1999). The conversion of NO_3_^−^ through the assimilation pathway must transport NO_3_^−^ into the cell. The cell dry weight and cell total nitrogen were increased along with the growth of *P. megaterium* NCT-2, while total nitrogen in supernatant was decreased. Meanwhile, the cell total nitrogen-^15^N atom% was the same as the marker NO_3_^−^-^15^N atom%. Therefore, assimilation is a mechanism for NO_3_^−^ transformation in *P. megaterium* NCT-2.

DNRA is known to occur in anaerobic or anaerobic microsites under aerobic conditions (Pandey et al., 2020). *P. megaterium* NCT-2 could utilize a small amount of NO_3_^−^ to generate NH_4_^+^ in anaerobic conditions, and NH_4_^+^-^15^N of the medium was diluted by unlabeled NH_4_^+^-N (Cheng et al., 2015). These proved that *P. megaterium* NCT-2 can transform NO_3_^−^ through DNRA. However, the NO_3_^−^ conversion amount of *P. megaterium* NCT-2 by DNRA was much less than the assimilation pathway. Furthermore, the N_2_O content and N_2_O-^15^N atom% were not changed by *P. megaterium* NCT-2. Previous studies have shown that if the strain utilizes ^15^NO_3_^−^ by denitrification, both the N_2_O content and N_2_O-^15^N atom% will increase (Castellanohinojosa et al., 2020). Therefore, *P. megaterium* NCT-2 cannot remove NO_3_^−^ by denitrification. In summary, NO_3_^−^ was mainly converted and utilized through the assimilation pathway in *P. megaterium* NCT-2.

Gene knockout can be used to predict the gene function by changing or shielding the gene (Marzan and Shimizu, 2011). It is well known that nitrate reductase catalyzes the reduction of NO_3_^−^ to NO_2_^−^, and nitrite reductase catalyzes the reduction of NO_2_^−^ to NH_4_^+^ in the assimilation pathway. Previous genome sequencing has also shown that the nitrate reductase genes (*nasB* and *nasC*) and nitrite reductase genes (*nasD* and *nasE*) in *P. megaterium* NCT-2 are involved in NO_3_^−^ reduction (Chu et al., 2017; Wang et al., 2020). Therefore, *nasC* and *nasD* genes were selected as the target genes for knockout. Previous study demonstrated that the mutant strains can transform NO_3_^−^ without affecting growth after knocking out the target gene, indicating that knocked-out genes are not key genes for NO_3_^−^ transformation (Wang et al., 2013). On the contrary, the mutant strains could not utilize NO_3_^−^, which proved that knockout genes are the key genes for NO_3_^−^ transformation (Wang et al., 2013). In this study, two mutant strains of *nasC* and *nasD* genes grew only slightly in NO_3_^−^ as a nitrogen source. The nitrate reductase and nitrite reductase are the key enzymes of NO_3_^−^ reduction (Morozkina and Zvyagilskaya, 2007). Thus, the knockout of NO_3_^−^ reduction genes inhibited the reduction of NO_3_^−^ into NO_2_^−^ and NH_4_^+^, which inhibited cell growth. Some studies have shown that *nasB* gene has the function of reducing nitrite in *Bacillus* (González et al., 2006). Therefore, there may be replacement genes for the *nasC* and *nasD* genes in *P. megaterium* NCT-2, which allow the strain to grow weakly. Furthermore, DNRA may occur in anaerobic microsites under aerobic conditions (Minick et al., 2016). Here, our results affirmed that *P. megaterium* NCT-2 can perform DNRA, such that it was able to metabolize a small amount of NO_3_^−^ through DNRA for cell growth.

The growth amount in the two complement strains of *nasC* and *nasD* genes was smaller than wild strain. The expression promoter of the *nasC* and *nasD* genes may be weaker in plasmid pWH1520 than in the wild strain, which could lead to a decrease in the *nasC* and *nasD* genes. Furthermore, the amount of nitrate transformation by *P. megaterium* NCT-2-△*nasC* mutant was obviously lower than that of the complement strain. This also proved that *nasC* gene is the key gene of NO_3_^−^ transformation. *P. megaterium* NCT-2 -△*nasD* mutant accumulated the most NO_2_^−^, while no NO_2_^−^ was accumulated in the complement strain. Thus, the knockout of *nasD* gene induced NO_2_^−^accumulation. In conclusion, *nasC* and *nasD* genes are the key genes for NO_3_^−^ reduction in *P. megaterium* NCT-2.

The current study suggests that the NO_3_^−^ metabolic pathway of *P. megaterium* NCT-2 is mainly an assimilation pathway. Nitrate assimilation is the main pathway of converting inorganic nitrogen into organic nitrogen and exists in a variety of organisms, including bacteria, yeast, and fungi (Damashek and Francis, 2018). These organisms with the function of assimilating nitrates provide nitrogen demand for other organisms, which has important biological significance.

### 4.2 The resistant mechanism of *Priestia megaterium* to salt stress

#### 4.2.1 Decomposition and metabolic processes

The utilization of nitrogen sources is inseparable from the synergistic effect of cellular metabolism. It is well known that amino acid metabolism is the basic metabolism of microorganisms. Sugar catabolism and energy metabolism are the main ways for organisms to obtain energy. ATP transporter is a protein family with transport functions (Canback et al., 2002). The rate-limiting enzyme genes or key genes of these processes in *P. megaterium* were significantly up-regulated by NO_3_^−^, which directly affects the metabolic capacity of cells (Sosa-Saavedra et al., 2001). *P. megaterium* NCT-2 accelerated the extracellular transport of nitrate and other nutrients for amino acid metabolism through the ATP transporter (Beis, 2015). Subsequently, substances (such as acetyl-CoA) were produced and entered into energy metabolism, including the TCA cycle, glycolysis, and oxidative phosphorylation (Kadenbach, 2018; Steffens et al., 2021). These processes produced large amounts of nucleotide and amino acid precursors for the expression of metabolic enzymes. Therefore, *P. megaterium* regulated metabolism and transport processes synergistically to promote transformation and absorption of nutrients in NO_3_^−^ as a nitrogen source. This is consistent with previous studies, which found that bacteria maintained growth by promoting basal and energy metabolism in stress conditions (Qiao et al., 2019).

In addition, bacteria use two-component systems as a means of adapting to their environment (Nguyen and Hong, 2008). Two-component system-related genes of *P. megaterium* were significantly down-regulated, especially histidine kinase. Histidine kinase is the core protein in the two-component system, which has kinase, phosphotransferase, and phosphatase activities (Nguyen and Hong, 2008). This implied that the NO_3_^−^ did not threaten the survival of *P. megaterium*, which could adapt to NO_3_^−^.

The key enzyme genes of ATP transport and oxidative phosphorylation pathways were significantly down-regulated in salt stress, which could reduce bacterial utilization of nutrients. This was consistent with the results of bacterial growth, where salt stress was found to interfere with the conversion and utilization of NO_3_^−^ by *P. megaterium*. However, four genes related to amino acid metabolism were found to have the largest up-regulation in salt stress. The importance of amino acid metabolism in adaptation to salt stress has been demonstrated in microorganisms and plants. Amino acids can be used as osmotic protectants to restore osmotic homeostasis in salt stress (Zhang et al., 2017). Therefore, these genes may play a key role in the response of *P. megaterium* to salt stress.

#### 4.2.2 Adaptation strategies of *Priestia megaterium* to salt stress

Salt stress has been reported to induce cell damage and oxidative damage in microorganisms. Microorganisms must control damage and repair themselves by regulating functional processes and activating interlocking defense functions (Bi et al., 2018). This study suggested that *P. megaterium* responded to salt stress through multiple strategies.

Spores are dormant bodies produced by bacteria in a certain environment and can remain viable for several years to decades. It is extremely resistant to high temperatures, ultraviolet light, and many toxic chemicals. The sporulation is catalyzed by a series of spore-forming proteins (Tan and Ramamurthi, 2014). The NO_3_^−^ and salt stress significantly up-regulated the main genes of the sporulation (Kim et al., 2006). Moreover, YjcZ family sporulation protein, *CotE,* and small acid-soluble spore protein genes were the most up-regulated genes in NO_3_^−^ as a nitrogen source. *CotE* and small acid-soluble spore protein genes are key genes for sporulation and protect DNA (Tan and Ramamurthi, 2014; Wetzel and Fischer, 2015). The function of the YjcZ family sporulation proteins is unclear. However, the results of this study suggested that they could have a key role in sporulation. Therefore, the spore formation may be a possible strategy of *P. megaterium* in response to salt stress. This could be caused by the nutrient deficiency of the strain and the stress of NO_2_^−^ in NO_3_^−^ as a nitrogen source. In most cases, spores can form in adverse conditions. However, some bacteria can only produce spores in rich nutrition and suitable conditions. For example, *Bacillus thuringiensis* must be cultured in adequate nutrition and suitable temperature to form spores in large numbers (Lv et al., 2019). Therefore, spores cannot be simply understood as a product of adverse conditions. However, regardless of growth conditions, bacteria are highly resistant to adverse conditions after forming spores.

Reactive oxygen species (ROS) are produced naturally during mitochondrial aerobic metabolism, which maintains a dynamic equilibrium in normal conditions (Yang and Lee, 2015b). However, ROS homeostasis is disrupted in salt stress (Yang and Lee, 2015a). Therefore, the first antioxidant mechanisms of *P. megaterium* NCT-2 will be activated, such as SOD, CAT, and universal stress protein (Kvint et al., 2003). Moreover, heat shock proteins can form the first line of defense against protein aggregation in stress responses (Carra et al., 2017). Poly-*γ*-glutamate synthase can help bacteria survive in salt stress and participate in detoxification (Candela and Fouet, 2006). This is consistent with previous studies, which found that microorganisms can resist salt stress by up-regulating stress proteins (Jiang et al., 2019). Therefore, the significant up-regulation of these genes may be the key strategy of *P. megaterium* in response to NO_3_^−^ and salt stress. In addition, TetRs monitor cellular dynamics and regulate genes, including osmotic stress and metabolic regulation (Deng et al., 2013). The SOS response can stop DNA replication and cell division to protect bacteria (Janion, 2008). The up-regulation of TetRs and SOS response-associated peptidase genes highlighted the important role of TetRs and SOS response in *P. megaterium* response to salt stress.

Furthermore, key genes associated with both vesicle formation and flagellar assembly were significantly upregulated in *P. megaterium* NCT-2 under nitrate and salt stress. First, the formation of bacterial vesicles is considered an adaptive mechanism. Some studies suggest that vesicles can alter cell buoyancy, encouraging cells to float to the liquid surface, which might represent a microenvironment with lower salinity or more favorable nutrients (Winter et al., 2018; Tan et al., 2021). Therefore, the upregulation of vesicle-related genes in *P. megaterium* NCT-2 under salt stress may aid in relocating to a more suitable living space. More critically, the upregulation of flagellar assembly provides a direct motile advantage for coping with salt stress. The primary function of flagella is to drive bacterial motility, enabling cells to actively seek nutrients and escape harmful conditions via chemotaxis (Winter et al., 2018; Tan et al., 2021). Under high-salt stress, this motility is crucial. Flagella-driven locomotion allows bacteria to actively escape local microenvironments with critically high salt concentrations and migrate toward zones more conducive to growth. Furthermore, salt stress causes drastic osmotic changes, and the activation of the flagellar system is likely linked to the perception of such environmental stress signals. Research indicates that flagellar gene expression is often regulated by complex networks that integrate various environmental cues, including osmotic pressure (Lertsethtakarn et al., 2011). Thus, the upregulation of flagellar assembly genes represents not merely an enhancement of motility but is likely an active adaptive response initiated upon sensing salt stress, aimed at mitigating it by altering the bacterium’s spatial position.

Therefore, we conclude that by increasing vesicle and flagella formation, *P. megaterium* NCT-2 enhances its ability to spatially locate and migrate to more favorable environments and promote nutrient uptake, collectively alleviating the damage caused by salt stress.

## 5 Conclusion

Improvement and tolerance mechanisms of *Priestia megaterium* NCT-2 to salt ions in secondary saline soil. The study found that the NCT-2 strain significantly reduced some salt ions. The largest amount of salt ions removed is NO_3_^−^, followed by Na^+^ and Cl^−^. In detail, *P. megaterium* was found to be able to use NO_3_^−^ as a nitrogen source mainly through the assimilation pathway. In addition, transcriptomics revealed that spore formation and germination, antioxidant stress, flagellar assembly, and vesicle formation are also the main strategies for *P. megaterium* to adapt to NO_3_^−^, Na^+^, and Cl^−^. Moreover, the current study also identified the candidate genes involved in NO_3_^−^ metabolism and salt stress response. Overall, a comprehensive analysis of metabolism and functional processes in *P. megaterium* revealed that the strain adapts to salt stress by transforming NO_3_^−^ and regulating self-tolerance. This study provided evidence that the strain can remove NO_3_^−^ from soil and water under salt stress. Furthermore, it also provided candidate genes for NO_3_^−^ removal and the resistance to salt stress, which will improve the function of the strain.

## Data Availability

The raw RNA-seq data generated in this study have been deposited in the NCBI Sequence Read Archive (SRA) under accession number PRJNA1367947 (SAMN53358798, SAMN53358799, SAMN53358800, SAMN53358801, SAMN53358802, SAMN53358803, SAMN53358804, SAMN53358805, SAMN53358806).

## Footnotes

1
www.majorbio.com

2
http://bowtie-bio.sourceforge.net/bowtie2/index.shtml

3
http://rfam.xfam.org/

4
http://bioconductor.org/packages/release/bioc/html/DESeq2.html

5
http://www.geneontology.org/

6
https://github.com/tanghaibao/GOatools

7
http://kobas.cbi.pku.edu.cn/home.do

8
www.genescloud.cn
