# Supplementary material for: Improvement and tolerance mechanisms of Priestia megaterium to salt ions
Source: Front Microbiol. 2025 Dec 18;16:1730703. doi: 10.3389/fmicb.2025.1730703 (PMC12756439; doi:10.3389/fmicb.2025.1730703)
Supplement: Supplementary file 1 [file Supplementary_file_1.docx]

Supplementary Material

**Supplementary Figures and Results**

**3.1 Construction of recombinant knockout vector**

First, the upper and lower homology arm fragments of the *nasC* (*nasC* L and *nasC* R) and *nasD* (*nasD* L and *nasD* R) genes were amplified using the *P. megaterium* NCT-2 genome as a template, and these genes length were about 300 bp (Supplementary Figure 1a). Secondly, pBR322 plasmid was used as template to amplify the Cm fragment of chloramphenicol resistance gene (660 bp) (Supplementary Figure 1). Thirdly, *nasC* L-Cm-*nasC* R fragments (1257 bp) and *nasD* L-Cm-*nasD* R fragment (1281 bp) were obtained by overlapping PCR (Supplementary Figure 1b and c). Fourth, recombinant plasmids (pHIS1525-*nasC* and pHIS1525-*nasD*) were obtained by digestion and ligation. After transformation, the *nasC* L-Cm-*nasC* R and *nasD* L-Cm-*nasD* R fragments were confirmed to be inserted into pHIS1525 plasmid by enzyme digestion and PCR amplification, respectively. This proved that the replacement knockout vectors pHIS1525-*nasC* L-Cm-*nasC* R and pHIS1525-*nasD* L-Cm-*nasD* R was successfully constructed (Supplementary Figure 1d).


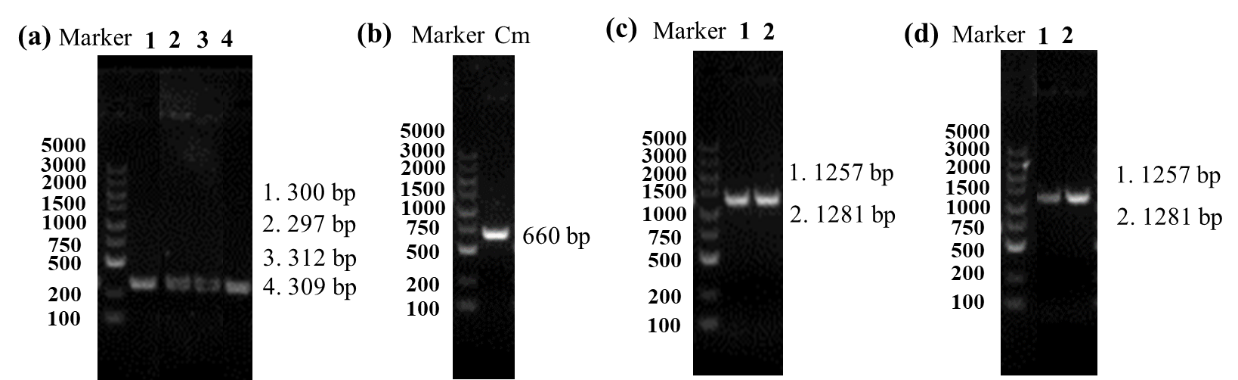


**Supplementary Figure 1** Electrophoresis diagram of recombinant knockout vector construction. (a) The upper and lower homology arms of *nasC* and *nasD* genes, 1. *NasC* upper homology arm, 2. *NasC* lower homology arm, 3. *NasD* upper homology arm, 4. *NasD* lower homology arm; (b) Amplification of the chloramphenicol resistance (CmR) gene cassette. The purified PCR product of the CmR gene was excised and purified for subsequent cloning into the knockout vector. (c) The upper and lower homology arms of *nasC* and *nasD* genes were respectively connected with *Cm* gene, 1. *NasC*L-Cm-*nasC*R, 2. *NasD*L-Cm-*nasD*R; (d) *NasC*L-Cm-*nasC*R and *nasD*L-Cm-*nasD*R fragments were respectively connected to the pHIS1525 vector, 1. *NasC*L-Cm-*nasC*R, 2. *NasD*L-Cm-*nasD*R.

**3.2 Knockdown validation of *nasC* and *nasD* genes in *P. megaterium* NCT-2 strain**

The knockout vectors pHIS1525-*nasC*L-Cm-*nasC*R and pHIS1525-*nasD*L-Cm-*nasD*R were transformed into *P. megaterium* NCT-2, respectively. Successfully transformed mutants were obtained by screening in resistant plates. The *P. megaterium* NCT-2 and two mutant strains were inserted into the Cm resistant plate. The results showed that *P. megaterium* NCT-2 could not grow, but the mutant strain could grow better, indicating that the Cm gene had been integrated into the mutant strain. Subsequently, PCR amplification was used to verify whether homologous exchange occurred on the genome. The length of the original nasC fragment was 2748 bp. If homologous recombination occurred at the nasC locus, the length of the fragment was 1257 bp. The length of the original *nasC* fragment was 2748 bp, and the length of the homologous recombination fragment was 1257 bp (Supplementary Figure 2). The length of the original *nasD* fragment was 3036 bp, and the length of the homologous recombination fragment was 1281 bp (Supplementary Figure 2). PCR results showed that two mutant strains had correct homologous recombination (Supplementary Figure 2). The *nasC* and *nasD* gene-deficient strains (*P. megaterium* NCT-2-△*nasC* and *P. megaterium* NCT-2-△*nasD*) were successfully constructed.


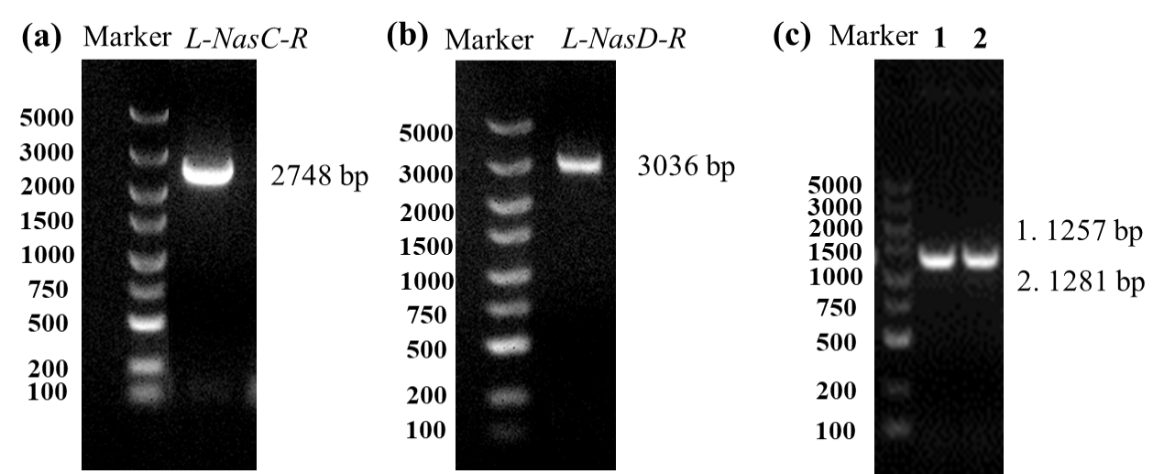


**Supplementary Figure 2** Electrophoresis tested gene knockout (*nasC* and *nasD*) in *P. megaterium* NCT-2. (a) *nasC* gene of *P. megaterium* NCT-2; (b) *nasD* gene of *P. megaterium* NCT-2; (c) *nasC* gene in *P. megaterium* NCT-2-△*nasC* mutant (1) and the *nasD* gene in *P. megaterium* NCT-2-△*nasD* mutant (2).

**3.3 Construction of *nasC and nasD* genes complement strains**

The *nasC* (with promoter), *nasD* (with promoter) and Km gene fragments were obtained by PCR amplification, and the gene lengths were 2170 bp, 3145 bp and 1035 bp, respectively (Supplementary Figure 3a). The *nasC*-Km (3205 bp) and the *nasD*-Km gene fragments (4180 bp) were obtained by overlapping PCR, respectively (Supplementary Figure 3b). Subsequently, the gene fragments and plasmid pWH1520 were digested and ligated to construct expression vectors of *nasC* (pWH1520-*nasC-*Km) and *nasD* (pWH1520-*nasD-*Km) genes, respectively (Supplementary Figure 3c). The recombinant vector pWH1520-*nasC-*Km was transformed into *P. megaterium* NCT-2-△*nasC* mutant, and pWH1520-*nasD-*Km was transformed into *P. megaterium* NCT-2-△*nasD* mutant. The complement strains of *nasC* (*P. megaterium* NCT-2-*nasC*-Km) and *nasD* (*P. megaterium* NCT-2-*nasC*-Km) genes were obtained.


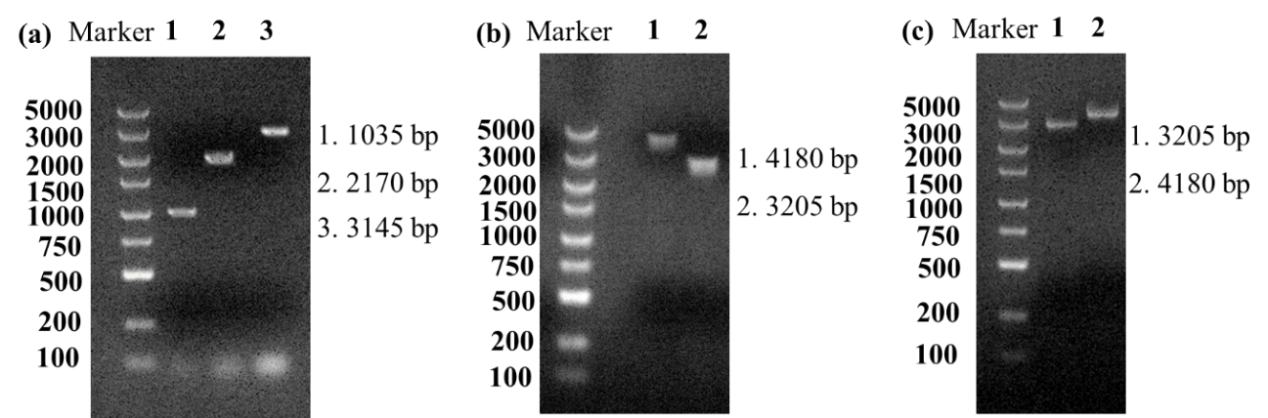


**Supplementary Figure 3** Electrophoresis of the recombination vector. (a) *NasC*, *nasD* and Km genes, 1. Km gene, 2. *NasC* gene and 3. *NasD* gene; (b) *NasC* and *nasD* genes were connected to Km gene respectively, 1. *NasC*-Km gene and 2. *NasD*-Km gene; (c) *NasC*-Km gene and *nasD*-Km gene were respectively connected to the vector pWH1520, 1. *NasC*-Km gene and 2. *NasD*-Km gene.

**3.4 Transformation and utilization of nitrate by *P. megaterium* NCT-2**

The transformation and utilization of nitrate by microorganisms is closely related to the functional genes of nitrogen metabolic pathway. Analysis revealed that nitrite reductase (*nasD* and *nasE*) and nitrate transporter (*nasA*) genes of *P. megaterium* NCT-2 were up-regulated by 2.50, 5.08 and 2.65 times in CK vs NCTa group, respectively (Supplementary Figure 4a). However, nitrate reductase genes (*nasB* and *nasC*) were down-regulated by 0.23 and 0.66 times, respectively (Supplementary Figure 4a). Therefore, this study speculated that the high expression of *nasD*, *nasE* and *nasA* genes is associated with nitrate metabolism. In NCTa vs NCTb group, *nasD*, *nasE*, *nasB*, *nasC* and *nasA* genes were down-regulated by 0.50, 0.42, 0.33, 0.28 and 0.20 times, respectively (Supplementary Figure 4b). This demonstrated that salt stress reduced the nitrate metabolic rate of *P. megaterium* NCT-2.

Glutamine synthase (*glnA*, EC: 6.3.1.2), glutamate synthase (*gltB*, EC: 1.4.1.13) and glutamate dehydrogenase (*gudB*, EC: 1.4.1.2; *gdhA*, EC: 1.4 .1.3) are key enzymes in glutamate metabolism and catalyze the production of glutamate from NH_4_^+^. In CK vs NCTa group, *glnA*, *gltB*, *gudB* and *gdhA* genes were respectively down-regulated by 0.74, 0.50, 0.81 and 0.76 times, but none of them were significant (Supplementary Figure 4a). This suggested that glutamate metabolism of *P. megaterium* NCT-2 could be slightly slowed in NO_3_^-^ as a nitrogen source compared with NH_4_^+^. In NCTa vs NCTb group, *glnA*, *gltB* and *gudB* genes were respectively up-regulated by 1.79, 1.23 and 4.80 times, while *gdhA* gene was down-regulated by 0.66 times (Supplementary Figure 4b). These results demonstrated that salt stress may promote glutamate metabolism of *P. megaterium* NCT-2.

**Supplementary Figure 4** Relative expression levels of nitrogen metabolic genes. (a) CK vs NCTa group; (b) NCTa vs NCTb group. Positive numbers were up-regulation, negative numbers were down-regulation.

The NO_3_^-^ reduction has been reported to occur in the cytoplasm. The NarK/NasA family of nitrate transporters are responsible for transporting NO_3_^-^ (Richardson et al., 2007). The NO_3_^-^ increased the expression of *nasA* gene in *P. megaterium* NCT-2 compared with NH_4_^+^, which a large amount of NO_3_^-^ might be transported into cells. The NO_3_^-^ is reduced to NO_2_^-^ by nitrate reductase (*nasB* and *nasC* genes), and then the NO_2_^-^ is rapidly reduced to NH_4_^+^ by nitrite reductase (*nasD* and *nasE* genes) (Damashek and Francis, 2018). Compared with NH_4_^+^ as a nitrogen source, NO_3_^-^ significantly down-regulated *nasB* and *nasC* genes of *P. megaterium* NCT-2. Studies have shown that the expression of nitrate reductase genes (*nasB* and *nasC*) is induced by NO_3_^-^ and NO_2_^-^ and inhibited by NH_4_^+^ (Luque-Almagro et al., 2011). The NH_4_^+^ production in nitrate metabolism may inhibit the *nasB* and *nasC* genes. However, NH_4_^+^ may have been completely consumed in NH_4_^+^ as a nitrogen source at the time of harvesting cells (48 h), and there was no inhibitory effect on the *nasB* and *nasC* genes. Moreover, it has been suggested that some genes may have multiple functions (Mhatre et al., 2020). In this study, GO and KEGG annotations showed that *nasB* and *nasC* genes in *P. megaterium* NCT-2 also had oxidoreductase functions. Therefore, these genes could be performed other functions in NH_4_^+^ as a nitrogen source, while their oxidoreductase action and expression were weakened in NO_3_^-^ as a nitrogen source. The nitrite reductase genes (*nasD* and *nasE*) of *P. megaterium* NCT-2 were upregulated in NO_3_^-^, which were beneficial to the utilization of NO_2_^-^. This result was consistent with previous studies, which found that the transcription level of bacterial nitrite reductase gene was higher in NO_3_^-^ than in NH_4_^+^ (Suzuki et al., 1995). Furthermore, the high expression of *nasD* and *nasE* genes may also be the self-detoxification of *P. megaterium*. These genes can be induced to reduce the toxic effects of intermediates and NO_2_^-^ (Mania et al., 2016), which has also been reported in *E. coli* (Corker and Poole, 2003).

Some stress affects the protein expression, which in turn interferes with the metabolic process. The nitrate transporter (*nasA*), nitrate reductase (*nasB* and *nasC*) and nitrite reductase genes (*nasD* and *nasE*) were down-regulated by salt stress in this study. Salt stress was also found to cause delayed growth in *P. megaterium*, which was consistent with the report by Nekolny and Chaloupka (Nekolny and Chaloupka, 2000). These suggested that salt stress could reduce the NO_3_^-^ conversion and utilization by *P. megaterium* NCT-2.

NH_4_^+^ production by NO_3_^-^ reduction is incorporated into biomolecules through glutamate metabolic pathways. The glutamine synthase (*glnA*), glutamate synthase (*gltB*) and glutamate dehydrogenase (*gudB* and *gdhA*) are the key enzymes (Boksha et al., 2018). These genes were down-regulated in NO_3_^-^ compared with NH_4_^+^. Microorganisms can directly use NH_4_^+^ to synthesize proteins in NH_4_^+^ medium. However, the reduction of NO_3_^-^ is a continuous process, and NH_4_^+^ is gradually produced and entered the glutamate metabolism in NO_3_^-^ medium (Yelamanchi et al., 2016). Therefore, these key enzyme genes of glutamate metabolism in *P. megaterium* were lower in NO_3_^-^ than in NH_4_^+^.

The *glnA*, *gltB* and *gudB* genes were up-regulated, while *gdhA* gene was down-regulated in salt stress. The up-regulation of *glnA* and *gltB* genes was beneficial to the rapid assimilation of NH_4_^+^. This also avoided cellular toxicity caused by excessive accumulation of NH_4_^+^. This study identified the differential expression of two glutamate dehydrogenases (up-regulated *gudB* gene, down-regulated *gdhA* gene). It is possible that this might be caused by the selective expression of certain isozymes. On the other hand, glutamate dehydrogenase can not only assimilate NH_4_^+^, but also can catalyze the release of NH_4_^+^ from glutamate (Chen et al., 2013). Therefore, when NH_4_^+^ assimilation is the main activity in strain, the expression of glutamate dehydrogenase responsible for the other activity is inhibited. Consequently, these findings shows that salt stress could promote glutamate metabolism of *P. megaterium* NCT-2.

**3.5 Validation of transcriptome results by RT-PCR**

The transcriptome results were validated by RT-PCR. RT-PCR determined the relative expression of 10 genes, including the key enzyme genes of nitrate assimilation pathway (*nasD*, *glnA*), glutamate metabolism genes (*gltB*, *gudB*, *gdhA*), catalase gene (*cat*), key genes for spore formation (*kinB*), isocitrate dehydrogenase (*icd*), 6-phosphogluconate dehydrogenase (*gnd*) and 16S gene. The results showed that the relative expression levels of *nasD*, *cat*, *icd*, *kinB* and *gnd* genes were significantly up-regulated by 4.5, 5.9, 5.02, 4.9 and 3.2 times in CK vs NCTa group, respectively. The relative expression levels of *glnA*, *gltB*, *gudB* and *gdhA* genes did not change significantly, which were 0.9, 0.93, 1.05 and 0.94, respectively (Supplementary Figure 5). In the NCTa vs NCTb group, the relative expression levels of *cat* and *kinB* genes were significantly up-regulated by 8.63 and 7.46 times, respectively (Supplementary Figure 5). The relative expression of *nasD* gene was significantly down-regulated by 0.56 times (Supplementary Figure 5). The relative expression levels of *glnA*, *gltB*, *gudB*, *gdhA*, *icd* and *gnd* genes did not change significantly, which were 1.1, 1.06, 1.09, 0.92, 0.93 and 0.95, respectively (Supplementary Figure 5). This result was basically consistent with the results of transcriptomics, and it proved that the results of transcriptome were true and reliable.

**Supplementary Figure 5** Relative expression levels of some differential genes. (a) CK vs NCTa comparison group; (b) NCTa vs NCTb comparison group. Note: The control is 1, greater than 1 for up-regulation, and less than 1 for down-regulation.

**Supplementary Table**

**Supplementary Table 1** List of primers for gene knockout

| Gene | Primer | Sequence (5’-3’) | Thermal profiles |
| --- | --- | --- | --- |
| *NasC*-L | F | TAGACAGAGGATGGGAAATATATGTCGGCG | 30 s at 95 °C, 40 cycles consisting of 5 s at 95 °C and 30 s at 60 °C, 15 s at 95 °C, 1 min at 60 °C and 15 s at 95 °C |
|  | R | CGTAGGTCACCCTTCTTTCCTATAATAAAT |  |
| *NasC-*R | F | GCGTCTTTATCTGCTAGAAATTTCGAATCT |  |
|  | R | TTAATAGCTGCTTCT TTTTACAGAAGAGCT |  |
| *NasD*-L | F | ACTACTGCTCATTTTTAATAGAAGTGATTT |  |
|  | R | CTTCCATTCCTCCCCTGGCGTCTATACCTG |  |
| *NasD*-R | F | TGCTGGGGGGAAATAAAGATGGTGAATAAA |  |
|  | R | AACAGACGCTTCGTACGTGTCTACACATCC |  |
| Cm | F | ATGGAGAAAAAAATCACTGGATATACCACC |  |
|  | R | TTACGCCCCGCCCTGCCACTCATCGCAGTA |  |
| *NasC*-L:: Cm:: *NasC*-R  overlap PCR | F1 | CGAGATCTTAGACAGAGGATGGGAAATATATGTCGGCG |  |
|  | R1 | GGAAAGAAGGGTGACCTACGCCAGTGATTTTTTTCTCCAT |  |
|  | F2 | GGAAAGAAGGGTGACCTACGATGGAGAAAAAAATCACTGG |  |
|  | R2 | AGTGGCAGGGCGGGGCGTAATTTCTAGCAGATAAAGACGC |  |
|  | F3 | AGTGGCAGGGCGGGGCGTAAGCGTCTTTATCTGCTAGAAA |  |
|  | R3 | CGGGATCCTTAATAGCTGCTTCTTTTTACAGAAGAGCT |  |
| *NasD*-L:: Cm:: *NasD*-R  Overlap PCR | F1 | CGAGATCTACTACTGCTCATTTTTAATAGAAGTGATTT |  |
|  | R1 | CGCCAGGGGAGGAATGGAAGCCAGTGATTTTTTTCTCCAT |  |
|  | F2 | CGCCAGGGGAGGAATGGAAGATGGAGAAAAAAATCACTGG |  |
|  | R2 | AGTGGCAGGGCGGGGCGTAAATCTTTATTTCCCCCCAGCA |  |
|  | F3 | AGTGGCAGGGCGGGGCGTAATGCTGGGGGGAAATAAAGAT |  |
|  | R3 | CGGGATCCAACAGACGCTTCGTACGTGTCTACACATCC |  |
| *NasC*-L:: Cm:: *NasC*-R | F | TAGACAGAGGATGGGAAATATATGTCGGCG |  |
|  | R | TTAATAGCTGCTTCT TTTTACAGAAGAGCT |  |
| *NasD*-L:: Cm:: *NasD*-R | F | ACTACTGCTCATTTTTAATAGAAGTGATTT |  |
|  | R | AACAGACGCTTCGTACGTGTCTACACATCC |  |

**Supplementary Table 2** Primers list for vector construction in gene replenishment

| Gene | Primer | Sequence (5’-3’) |
| --- | --- | --- |
| *NasC*（*Kpn*I） | F | GGGGTACCCCGAAAGAAGGGTGACCTACGATGACTGAGAT |
|  | R | GGATCAAGAGCTACCAACTCTTAATAGCTGCTTCTTTTTA |
| *NasD*（*Kpn*I） | F | GGGGTACCCCAAAGACAAGCCCTATTAAATGTGGAATAGG |
|  | R | GGATCAAGAGCTACCAACTCTTATGATGTGACAACTGTTT |
| *NasC*:: Km  overlap PCR（*BamH*I） | F | TAAAAAGAAGCAGCTATTAAGAGTTGGTAGCTCTTGATCC |
|  | R | CGGGATCTCGTTAGAAAAACTCATCGAGCATCAAATGAAAC |
| *NasD*:: Km  overlap PCR（*BamH*I） | F | AAACAGTTGTCACATCATAAGAGTTGGTAGCTCTTGATCC |
|  | R | CGGGATCTCGTTAGAAAAACTCATCGAGCATCAAATGAAAC |

**Supplementary Table 3** Primers used in RT-PCR

| Gene name | Encoding protein | Primer sequence (5 '-3') |
| --- | --- | --- |
| 16S rRNA | Reference gene | F: GGGCGGAAACCCTCTAACAC |
|  |  | S: GAAAGCGTGGGGAGCAAA |
| *nasD* | Nitrite reductase | F: TTCTTCCGATTCCAGGGTC |
|  |  | S: AGTCCGCCTCCAATAACC |
| *glnA* | Glutamine synthase | F: ACCGTAGCCCATTAATCCGT |
|  |  | S: GGGTCTACGCTACGTACCTC |
| *gltB* | Glutamate synthase | F: AACAGTTTGTTTCGGACGCA |
|  |  | S: CTCAGACCAAGGCTCTGGAA |
| *gudB* | Glutamate dehydrogenase | F: TTTCACCCATCTGTCAACGC |
|  |  | S: AAATCAGCACCGCCTTTACC |
| *gdhA* | Glutamate dehydrogenase | F: CTTGATCGACGCGACAGTTT |
|  |  | S: CGTAGCTTCAAGCGTAGTCG |
| *icd* | Isocitrate dehydrogenase | F: TCTCTAAACGTGGCATTGCG |
|  |  | S: GGGCGCTTAATTGGAGAAGG |
| *gnd* | 6-phosphogluconate dehydrogenase | F: TATGGTTCCGGCTGGAGAAG |
|  |  | S: TTTCTTGCGCCTTCCATTCC |
| *cat* | Catalase | F: AAACGGAGCAAGTCGCATTT |
|  |  | S: GGCAGCTGCAAGTAGTTTGT |
| *kinB* | KinB signaling pathway activator | F: GCATCACTTTGGAAGTCGGT |
|  |  | S: TGCATACGCCACTAACAACC |

**Supplementary Table 4** Statistics of data volume after quality control

| Sample Name | Raw reads | Raw Bases (bp) | Raw Error Rate (%) | Raw Q20 (%) | Raw Q30 (%) |
| --- | --- | --- | --- | --- | --- |
| CK1 | 43176616 | 6519669016 | 0.0264 | 97.31 | 92.99 |
| CK2 | 44340398 | 6695400098 | 0.026 | 97.44 | 93.46 |
| CK3 | 42894450 | 6477061950 | 0.0256 | 97.63 | 93.76 |
| NCTa1 | 51100112 | 7716116912 | 0.0259 | 97.48 | 93.55 |
| NCTa2 | 45006992 | 6796055792 | 0.0261 | 97.32 | 93.38 |
| NCTa3 | 47849168 | 7225224368 | 0.0259 | 97.5 | 93.49 |
| NCTb1 | 45037802 | 6800708102 | 0.026 | 97.46 | 93.36 |
| NCTb2 | 44607074 | 6735668174 | 0.0261 | 97.39 | 93.29 |
| NCTb3 | 40938786 | 6181756686 | 0.0263 | 97.31 | 93.15 |

**Supplementary Table 5** Significantly up-regulated differential genes in CK vs NCTa comparison group

| Gene_id | Gene name | Gene description | FC | Log2FC |
| --- | --- | --- | --- | --- |
| OEA_RS03500 | OEA_RS03500 | cysteine synthase family protein | 23.27 | 4.54 |
| OEA_RS10650 | OEA_RS10650 | GlsB/YeaQ/YmgE family stress response membrane protein | 19.01 | 4.25 |
| OEA_RS19265 | galU | UTP--glucose-1-phosphate uridylyltransferase GalU | 6.38 | 2.67 |
| OEA_RS19280 | OEA_RS19280 | spore germination protein | 58.85 | 5.88 |
| OEA_RS03795 | OEA_RS03795 | spore protein YqfQ | 105.78 | 6.72 |
| OEA_RS03520 | sigK | RNA polymerase sporulation sigma factor SigK | 100.52 | 6.65 |
| OEA_RS03505 | OEA_RS03505 | bifunctional cystathionine gamma-lyase/homocysteine desulfhydrase | 25.22 | 4.66 |
| OEA_RS10995 | aroA | 3-phosphoshikimate 1-carboxyvinyltransferase | 9.05 | 3.18 |
| OEA_RS03230 | safA | SafA/ExsA family spore coat assembly protein | 50.87 | 5.67 |
| OEA_RS03240 | OEA_RS03240 | spore cortex protein | 210.88 | 7.72 |
| OEA_RS10985 | aroC | chorismate synthase | 18.50 | 4.21 |
| OEA_RS19045 | OEA_RS19045 | glycine betaine ABC transporter substrate-binding protein | 52.67 | 5.72 |
| OEA_RS19050 | OEA_RS19050 | proline/glycine betaine ABC transporter permease | 45.87 | 5.52 |
| OEA_RS19055 | OEA_RS19055 | betaine/proline/choline family ABC transporter ATP-binding protein | 28.38 | 4.83 |
| OEA_RS19060 | OEA_RS19060 | aromatic ring-hydroxylating dioxygenase subunit alpha | 33.04 | 5.05 |
| OEA_RS19065 | solA | N-methyl-L-tryptophan oxidase | 30.48 | 4.93 |
| OEA_RS19070 | OEA_RS19070 | FAD-dependent oxidoreductase | 13.08 | 3.71 |
| OEA_RS10380 | qoxB | cytochrome aa3 quinol oxidase subunit I | 4.37 | 2.13 |
| OEA_RS10375 | qoxA | cytochrome aa3 quinol oxidase subunit II | 4.34 | 2.12 |
| OEA_RS19605 | OEA_RS19605 | spore coat protein | 7.07 | 2.82 |
| OEA_RS24655 | OEA_RS24655 | gamma-type small acid-soluble spore protein | 195.72 | 7.61 |
| OEA_RS19640 | OEA_RS19640 | YhcN/YlaJ family sporulation lipoprotein | 127.41 | 6.99 |
| OEA_RS19680 | OEA_RS19680 | spore coat protein | 33.54 | 5.07 |
| OEA_RS09900 | OEA_RS09900 | hypothetical protein | 59.19 | 5.89 |
| OEA_RS24460 | yhbH | sporulation protein YhbH | 45.25 | 5.50 |
| OEA_RS24435 | thiC | phosphomethylpyrimidine synthase ThiC | 5.04 | 2.33 |
| OEA_RS11065 | OEA_RS11065 | copper resistance protein | 4.51 | 2.17 |
| OEA_RS03980 | OEA_RS03980 | Spx/MgsR family RNA polymerase-binding regulatory protein | 9.65 | 3.27 |
| OEA_RS04035 | gcvT | glycine cleavage system aminomethyltransferase GcvT | 13.83 | 3.79 |
| OEA_RS04040 | OEA_RS04040 | aminomethyl-transferring glycine dehydrogenase subunit GcvPA | 13.09 | 3.71 |
| OEA_RS04045 | OEA_RS04045 | glycine dehydrogenase subunit 2 | 11.79 | 3.56 |
| OEA_RS04075 | OEA_RS04075 | patatin-like phospholipase family protein | 11.77 | 3.56 |
| OEA_RS12635 | OEA_RS12635 | superoxide dismutase | 197.68 | 7.63 |
| OEA_RS02120 | OEA_RS02120 | DNA starvation/stationary phase protection protein | 10.51 | 3.39 |
| OEA_RS02140 | OEA_RS02140 | ABC transporter permease | 180.84 | 7.50 |
| OEA_RS02145 | OEA_RS02145 | ABC transporter ATP-binding protein | 201.90 | 7.66 |
| OEA_RS02150 | OEA_RS02150 | ABC transporter substrate-binding protein | 127.31 | 6.99 |
| OEA_RS26460 | yabG | sporulation peptidase YabG | 12.78 | 3.68 |
| OEA_RS17185 | OEA_RS17185 | universal stress protein | 24.02 | 4.59 |
| OEA_RS26450 | OEA_RS26450 | small%2C acid-soluble spore protein%2C alpha/beta type | 136.92 | 7.10 |
| OEA_RS17140 | OEA_RS17140 | 2-oxo acid dehydrogenase subunit E2 | 6.77 | 2.76 |
| OEA_RS17050 | OEA_RS17050 | universal stress protein | 8.80 | 3.14 |
| OEA_RS01835 | cobA | uroporphyrinogen-III C-methyltransferase | 5.47 | 2.45 |
| OEA_RS26415 | OEA_RS26415 | 50S ribosomal protein L25/general stress protein Ctc | 7.12 | 2.83 |
| OEA_RS17265 | fsa | fructose-6-phosphate aldolase | 27.10 | 4.76 |
| OEA_RS25890 | pdaB | polysaccharide deacetylase family sporulation protein PdaB | 20.28 | 4.34 |
| OEA_RS02870 | gerE | spore germination protein GerE | 386.79 | 8.60 |
| OEA_RS11325 | OEA_RS11325 | spore germination protein | 45.67 | 5.51 |
| OEA_RS18880 | OEA_RS18880 | alpha/beta-type small acid-soluble spore protein | 96.42 | 6.59 |
| OEA_RS18930 | OEA_RS18930 | amino acid permease | 29.23 | 4.87 |
| OEA_RS18950 | OEA_RS18950 | aldehyde dehydrogenase family protein | 4.66 | 2.22 |
| OEA_RS03075 | spoVID | stage VI sporulation protein D | 88.66 | 6.47 |
| OEA_RS02670 | OEA_RS02670 | glyceraldehyde-3-phosphate dehydrogenase | 11.66 | 3.54 |
| OEA_RS17515 | OEA_RS17515 | alpha/beta-type small acid-soluble spore protein | 173.26 | 7.44 |
| OEA_RS06120 | OEA_RS06120 | acetyltransferase | 5.00 | 2.32 |
| OEA_RS23160 | OEA_RS23160 | peptide ABC transporter substrate-binding protein | 18.69 | 4.22 |
| OEA_RS06250 | ablA | lysine 2%2C3-aminomutase | 42.93 | 5.42 |
| OEA_RS06110 | OEA_RS06110 | aspartate aminotransferase family protein | 4.55 | 2.18 |
| OEA_RS08260 | OEA_RS08260 | alpha/beta-type small acid-soluble spore protein | 390.91 | 8.61 |
| OEA_RS05910 | cotE | outer spore coat protein CotE | 480.74 | 8.91 |
| OEA_RS08045 | OEA_RS08045 | glutathione-dependent formaldehyde dehydrogenase | 104.22 | 6.70 |
| OEA_RS21630 | OEA_RS21630 | capsular polysaccharide biosynthesis protein | 11.61 | 3.54 |
| OEA_RS23185 | OEA_RS23185 | peptide ABC transporter substrate-binding protein | 6.73 | 2.75 |
| OEA_RS21635 | OEA_RS21635 | CapA family protein | 12.24 | 3.61 |
| OEA_RS21645 | pgsB | poly-gamma-glutamate synthase PgsB | 35.54 | 5.15 |
| OEA_RS22040 | rbsK | ribokinase | 21.52 | 4.43 |
| OEA_RS07600 | OEA_RS07600 | alpha/beta-type small acid-soluble spore protein | 228.04 | 7.83 |
| OEA_RS22770 | speE | polyamine aminopropyltransferase | 211.42 | 7.72 |
| OEA_RS06930 | ggt | gamma-glutamyltransferase | 66.03 | 6.05 |
| OEA_RS08270 | OEA_RS08270 | gamma-type small acid-soluble spore protein | 27.32 | 4.77 |
| OEA_RS22850 | OEA_RS22850 | general stress protein | 4.93 | 2.30 |
| OEA_RS07535 | OEA_RS07535 | alpha/beta-type small acid-soluble spore protein | 110.97 | 6.79 |
| OEA_RS05785 | OEA_RS05785 | ClpP family protease | 15.05 | 3.91 |
| OEA_RS24005 | OEA_RS24005 | SpoVR family protein | 87.30 | 6.45 |
| OEA_RS23900 | OEA_RS23900 | alpha/beta-type small acid-soluble spore protein | 88.09 | 6.46 |
| OEA_RS04780 | spoIVA | stage IV sporulation protein A | 42.42 | 5.41 |
| OEA_RS24080 | OEA_RS24080 | L-lactate dehydrogenase | 32.51 | 5.02 |
| OEA_RS24255 | OEA_RS24255 | alpha/beta-type small acid-soluble spore protein | 35.05 | 5.13 |
| OEA_RS19865 | coxB | cytochrome c oxidase subunit II | 4.41 | 2.14 |
| OEA_RS19870 | OEA_RS19870 | protoheme IX farnesyltransferase | 4.38 | 2.13 |
| OEA_RS04540 | OEA_RS04540 | spore germination protein | 19.74 | 4.30 |
| OEA_RS09680 | OEA_RS09680 | AraC family transcriptional regulator | 9.51 | 3.25 |
| OEA_RS04435 | OEA_RS04435 | CoA transferase subunit B | 5.45 | 2.45 |
| OEA_RS23385 | pruA | L-glutamate gamma-semialdehyde dehydrogenase | 16.89 | 4.08 |
| OEA_RS21055 | OEA_RS21055 | polysaccharide biosynthesis protein | 9.49 | 3.25 |
| OEA_RS21060 | galU | UTP--glucose-1-phosphate uridylyltransferase GalU | 7.48 | 2.90 |
| OEA_RS09185 | OEA_RS09185 | YjcZ family sporulation protein | 172.16 | 7.43 |
| OEA_RS09135 | OEA_RS09135 | fructose-1%2C6-bisphosphatase | 4.48 | 2.16 |
| OEA_RS20560 | OEA_RS20560 | alpha/beta-type small acid-soluble spore protein | 18.92 | 4.24 |
| OEA_RS23420 | asnB | asparagine synthase (glutamine-hydrolyzing) | 58.91 | 5.88 |
| OEA_RS00730 | OEA_RS00730 | M23 family metallopeptidase | 7.80 | 2.96 |
| OEA_RS00695 | OEA_RS00695 | F0F1 ATP synthase subunit gamma | 4.11 | 2.04 |
| OEA_RS15810 | OEA_RS15810 | squalene--hopene cyclase | 99.75 | 6.64 |
| OEA_RS13260 | OEA_RS13260 | sirohydrochlorin chelatase | 4.62 | 2.21 |
| OEA_RS31445 | OEA_RS31445 | alpha/beta-type small acid-soluble spore protein | 12.81 | 3.68 |
| OEA_RS13255 | cobJ | precorrin-3B C(17)-methyltransferase | 5.19 | 2.38 |
| OEA_RS01545 | OEA_RS01545 | sporulation protein | 22.10 | 4.47 |
| OEA_RS01100 | hisB | imidazoleglycerol-phosphate dehydratase HisB | 7.05 | 2.82 |
| OEA_RS01095 | hisD | histidinol dehydrogenase | 8.15 | 3.03 |
| OEA_RS01090 | OEA_RS01090 | ATP phosphoribosyltransferase | 8.57 | 3.10 |
| OEA_RS01020 | OEA_RS01020 | proline dehydrogenase | 8.65 | 3.11 |
| OEA_RS01190 | gap | type I glyceraldehyde-3-phosphate dehydrogenase | 5.38 | 2.43 |
| OEA_RS13545 | OEA_RS13545 | Hsp20/alpha crystallin family protein | 366.46 | 8.52 |
| OEA_RS14110 | OEA_RS14110 | alpha/beta-type small acid-soluble spore protein | 266.59 | 8.06 |
| OEA_RS13585 | OEA_RS13585 | small acid-soluble spore protein Tlp | 57.74 | 5.85 |
| OEA_RS31180 | OEA_RS31180 | alpha/beta-type small acid-soluble spore protein | 230.58 | 7.85 |
| OEA_RS14950 | OEA_RS14950 | LysR family transcriptional regulator | 7.82 | 2.97 |
| OEA_RS01375 | OEA_RS01375 | efflux RND transporter permease subunit | 5.48 | 2.45 |
| OEA_RS15565 | splB | spore photoproduct lyase | 14.60 | 3.87 |
| OEA_RS00285 | OEA_RS00285 | cytochrome ubiquinol oxidase subunit I | 11.58 | 3.53 |
| OEA_RS00290 | cydB | cytochrome d ubiquinol oxidase subunit II | 9.80 | 3.29 |
| OEA_RS00415 | gerQ | spore coat protein GerQ | 130.78 | 7.03 |
| OEA_RS31165 | OEA_RS31165 | cell wall hydrolase | 11.07 | 3.47 |
| OEA_RS14720 | OEA_RS14720 | valine--pyruvate transaminase | 7.89 | 2.98 |
| OEA_RS00420 | OEA_RS00420 | cell wall hydrolase | 131.22 | 7.04 |
| OEA_RS00295 | cydD | thiol reductant ABC exporter subunit CydD | 10.42 | 3.38 |
| OEA_RS15055 | OEA_RS15055 | peptidase M4 family protein | 180.53 | 7.50 |
| OEA_RS15045 | fsa | fructose-6-phosphate aldolase | 15.39 | 3.94 |
| OEA_RS01575 | yutH | spore coat protein YutH | 7.54 | 2.92 |
| OEA_RS01105 | hisH | imidazole glycerol phosphate synthase subunit HisH | 5.92 | 2.56 |
| OEA_RS05495 | fliF | flagellar basal body M-ring protein FliF | 59.80 | 5.90 |
| OEA_RS04710 | sleB | spore cortex-lytic enzyme | 55.22 | 5.79 |
| OEA_RS20905 | cobA | uroporphyrinogen-III C-methyltransferase | 4.08 | 2.03 |
| OEA_RS19125 | OEA_RS19125 | iron-hydroxamate ABC transporter substrate-binding protein | 4.23 | 2.08 |
| OEA_RS01085 | OEA_RS01085 | ATP phosphoribosyltransferase regulatory subunit | 7.07 | 2.82 |
| OEA_RS06235 | OEA_RS06235 | 3-oxoacid CoA-transferase subunit B | 26.77 | 4.74 |
| OEA_RS06810 | OEA_RS06810 | ABC transporter ATP-binding protein | 4.81 | 2.27 |
| OEA_RS02495 | ytfJ | sporulation protein YtfJ | 8.73 | 3.13 |
| OEA_RS04430 | OEA_RS04430 | CoA transferase subunit A | 5.58 | 2.48 |
| OEA_RS04145 | spoIIIAG | stage III sporulation protein AG | 24.66 | 4.62 |
| OEA_RS13280 | cbiE | precorrin-6y C5%2C15-methyltransferase (decarboxylating) subunit CbiE | 4.61 | 2.21 |
| OEA_RS02165 | pckA | phosphoenolpyruvate carboxykinase (ATP) | 5.65 | 2.50 |
| OEA_RS16640 | OEA_RS16640 | Hsp20/alpha crystallin family protein | 61.28 | 5.94 |
| OEA_RS10115 | OEA_RS10115 | siderophore ABC transporter substrate-binding protein | 4.19 | 2.07 |
| OEA_RS01840 | OEA_RS01840 | sirohydrochlorin chelatase | 4.07 | 2.02 |
| OEA_RS09875 | OEA_RS09875 | AraC family transcriptional regulator | 9.53 | 3.25 |
| OEA_RS22980 | OEA_RS22980 | sporulation protein | 302.40 | 8.24 |
| OEA_RS04525 | spoVAD | stage V sporulation protein AD | 73.08 | 6.19 |
| OEA_RS25175 | OEA_RS25175 | BMP family ABC transporter substrate-binding protein | 7.86 | 2.97 |
| OEA_RS19905 | OEA_RS19905 | YhcN/YlaJ family sporulation lipoprotein | 52.49 | 5.71 |
| OEA_RS23890 | ugpC | sn-glycerol-3-phosphate ABC transporter ATP-binding protein UgpC | 4.80 | 2.26 |
| OEA_RS11005 | OEA_RS11005 | FAD-dependent oxidoreductase | 4.64 | 2.21 |
| OEA_RS13550 | OEA_RS13550 | small acid-soluble spore protein P | 301.33 | 8.24 |
| OEA_RS10340 | cadA | cadmium-translocating P-type ATPase | 4.48 | 2.16 |
| OEA_RS00300 | cydC | thiol reductant ABC exporter subunit CydC | 7.19 | 2.85 |
| OEA_RS26390 | spoVT | stage V sporulation protein T | 78.47 | 6.29 |
| OEA_RS10125 | OEA_RS10125 | manganese catalase family protein | 14.66 | 3.87 |
| OEA_RS33185 | OEA_RS33185 | alpha/beta-type small acid-soluble spore protein | 14.21 | 3.83 |
| OEA_RS04535 | OEA_RS04535 | stage V sporulation protein AE | 37.12 | 5.21 |
| OEA_RS04150 | OEA_RS04150 | SpoIIIAH-like family protein | 4.88 | 2.29 |
| OEA_RS02950 | OEA_RS02950 | sporulation protein | 4.75 | 2.25 |
| OEA_RS11845 | OEA_RS11845 | ABC transporter ATP-binding protein | 11.47 | 3.52 |
| OEA_RS06935 | rocF | arginase | 22.89 | 4.52 |
| OEA_RS02700 | OEA_RS02700 | sporulation protein | 11.88 | 3.57 |
| OEA_RS18285 | OEA_RS18285 | H-type small acid-soluble spore protein | 6.22 | 2.64 |
| OEA_RS13290 | cobM | precorrin-4 C(11)-methyltransferase | 5.32 | 2.41 |
| OEA_RS23440 | OEA_RS23440 | spore germination protein | 154.97 | 7.28 |
| OEA_RS01110 | hisA | 1-(5-phosphoribosyl)-5-[(5-phosphoribosylamino)methylideneamino]imidazole-4-carboxamide isomerase | 4.31 | 2.11 |
| OEA_RS13270 | OEA_RS13270 | precorrin-8X methylmutase | 4.32 | 2.11 |
| OEA_RS23465 | OEA_RS23465 | spore germination protein | 158.15 | 7.31 |
| OEA_RS03250 | OEA_RS03250 | bypass-of-forespore protein C | 4.51 | 2.17 |
| OEA_RS08050 | OEA_RS08050 | spore coat protein | 92.98 | 6.54 |
| OEA_RS22985 | OEA_RS22985 | YjcZ family sporulation protein | 856.49 | 9.74 |
| OEA_RS06815 | OEA_RS06815 | ABC transporter permease/substrate-binding protein | 5.01 | 2.32 |
| OEA_RS11730 | OEA_RS11730 | DUF4183 domain-containing protein | 16.73 | 4.06 |
| OEA_RS20990 | OEA_RS20990 | SDR family NAD(P)-dependent oxidoreductase | 4.75 | 2.25 |
| OEA_RS10195 | OEA_RS10195 | ATP-binding protein | 4.58 | 2.19 |
| OEA_RS25760 | OEA_RS25760 | BglG family transcription antiterminator | 8.32 | 3.06 |
| OEA_RS03960 | OEA_RS03960 | peptidase M14 | 16.53 | 4.05 |
| OEA_RS24685 | OEA_RS24685 | small%2C acid-soluble spore protein K | 388.37 | 8.60 |
| OEA_RS23380 | OEA_RS23380 | ornithine--oxo-acid transaminase | 6.95 | 2.80 |
| OEA_RS23190 | OEA_RS23190 | dipeptide ABC transporter ATP-binding protein | 4.79 | 2.26 |
| OEA_RS11790 | OEA_RS11790 | 2OG-Fe(II) oxygenase | 5.30 | 2.40 |
| OEA_RS04140 | spoIIIAF | stage III sporulation protein AF | 13.27 | 3.73 |
| OEA_RS01520 | yunB | sporulation protein YunB | 4.57 | 2.19 |
| OEA_RS33230 | OEA_RS33230 | FAD-dependent oxidoreductase | 5.81 | 2.54 |
| OEA_RS11530 | OEA_RS11530 | polyamine aminopropyltransferase | 25.07 | 4.65 |
| OEA_RS12010 | OEA_RS12010 | carbohydrate ABC transporter permease | 26.14 | 4.71 |
| OEA_RS09890 | OEA_RS09890 | spore coat associated protein CotJA | 158.93 | 7.31 |
| OEA_RS23460 | OEA_RS23460 | spore germination protein GerPE | 53.27 | 5.74 |
| OEA_RS11310 | OEA_RS11310 | Hsp20/alpha crystallin family protein | 64.22 | 6.00 |
| OEA_RS03155 | OEA_RS03155 | M23 family metallopeptidase | 9.97 | 3.32 |
| OEA_RS23155 | OEA_RS23155 | ABC transporter permease | 19.29 | 4.27 |
| OEA_RS06955 | OEA_RS06955 | GerAB/ArcD/ProY family transporter | 29.06 | 4.86 |
| OEA_RS02745 | sspI | small acid-soluble spore protein SspI | 411.54 | 8.68 |
| OEA_RS10245 | OEA_RS10245 | cytochrome c oxidase assembly protein | 37.97 | 5.25 |
| OEA_RS23445 | OEA_RS23445 | spore germination protein | 85.97 | 6.43 |
| OEA_RS08035 | OEA_RS08035 | spore coat protein | 46.34 | 5.53 |
| OEA_RS02085 | OEA_RS02085 | cytochrome ubiquinol oxidase subunit I | 16.27 | 4.02 |
| OEA_RS13285 | cobI | precorrin-2 C(20)-methyltransferase | 5.56 | 2.47 |
| OEA_RS13265 | cobK | precorrin-6A reductase | 4.45 | 2.15 |
| OEA_RS04510 | OEA_RS04510 | stage V sporulation protein AA | 65.42 | 6.03 |
| OEA_RS15025 | sspL | small%2C acid-soluble spore protein L | 143.32 | 7.16 |
| OEA_RS22030 | OEA_RS22030 | sugar ABC transporter ATP-binding protein | 13.02 | 3.70 |
| OEA_RS09710 | OEA_RS09710 | YjcZ family sporulation protein | 280.51 | 8.13 |
| OEA_RS05500 | fliG | flagellar motor switch protein FliG | 17.87 | 4.16 |
| OEA_RS23920 | gpmA | 2%2C3-diphosphoglycerate-dependent phosphoglycerate mutase | 22.22 | 4.47 |
| OEA_RS11320 | OEA_RS11320 | spore germination protein | 16.96 | 4.08 |
| OEA_RS03360 | pstS | phosphate ABC transporter substrate-binding protein PstS family protein | 4.56 | 2.19 |
| OEA_RS09225 | OEA_RS09225 | spore germination protein | 182.61 | 7.51 |
| OEA_RS13575 | OEA_RS13575 | acid-soluble spore protein N | 4.76 | 2.25 |
| OEA_RS24875 | OEA_RS24875 | D-2-hydroxyacid dehydrogenase | 4.28 | 2.10 |
| OEA_RS21565 | OEA_RS21565 | 6-phospho-beta-glucosidase | 6.08 | 2.60 |
| OEA_RS19530 | ugpC | sn-glycerol-3-phosphate ABC transporter ATP-binding protein UgpC | 36.42 | 5.19 |
| OEA_RS00675 | atpE | F0F1 ATP synthase subunit C | 4.29 | 2.10 |
| OEA_RS13555 | OEA_RS13555 | small acid-soluble spore protein O | 224.36 | 7.81 |
| OEA_RS16020 | OEA_RS16020 | superoxide dismutase family protein | 5.55 | 2.47 |
| OEA_RS04115 | spoIIIAA | stage III sporulation protein AA | 12.72 | 3.67 |
| OEA_RS23795 | OEA_RS23795 | YjcZ family sporulation protein | 359.57 | 8.49 |
| OEA_RS05480 | flgB | flagellar basal body rod protein FlgB | 50.27 | 5.65 |
| OEA_RS12015 | OEA_RS12015 | carbohydrate ABC transporter substrate-binding protein | 10.44 | 3.38 |
| OEA_RS17510 | OEA_RS17510 | alpha/beta-type small acid-soluble spore protein | 272.07 | 8.09 |
| OEA_RS03295 | spoVB | stage V sporulation protein B | 12.53 | 3.65 |
| OEA_RS19535 | OEA_RS19535 | extracellular solute-binding protein | 13.52 | 3.76 |
| OEA_RS09895 | OEA_RS09895 | spore coat protein CotJB | 112.25 | 6.81 |
| OEA_RS02465 | OEA_RS02465 | alpha/beta-type small acid-soluble spore protein | 146.34 | 7.19 |
| OEA_RS15465 | OEA_RS15465 | YjcZ family sporulation protein | 76.89 | 6.26 |
| OEA_RS05750 | OEA_RS05750 | YlmC/YmxH family sporulation protein | 25.78 | 4.69 |
| OEA_RS16105 | OEA_RS16105 | sulfate ABC transporter substrate-binding protein | 7.60 | 2.93 |
| OEA_RS17460 | OEA_RS17460 | cell wall hydrolase | 41.13 | 5.36 |
| OEA_RS12705 | yjcZ | sporulation protein YjcZ | 190.34 | 7.57 |
| OEA_RS22035 | rbsD | D-ribose pyranase | 27.59 | 4.79 |
| OEA_RS13350 | OEA_RS13350 | response regulator transcription factor | 5.65 | 2.50 |
| OEA_RS03160 | OEA_RS03160 | stage IV sporulation protein FB | 7.80 | 2.96 |
| OEA_RS23520 | OEA_RS23520 | M48 family metallopeptidase | 4.19 | 2.07 |
| OEA_RS03695 | yqfC | sporulation protein YqfC | 25.10 | 4.65 |
| OEA_RS16040 | pfkB | 1-phosphofructokinase | 7.90 | 2.98 |
| OEA_RS04520 | spoVAC | stage V sporulation protein AC | 69.86 | 6.13 |
| OEA_RS18305 | OEA_RS18305 | glucose dehydrogenase | 4.19 | 2.07 |
| OEA_RS02890 | OEA_RS02890 | ABC transporter ATP-binding protein | 54.53 | 5.77 |
| OEA_RS06205 | OEA_RS06205 | YjcZ family sporulation protein | 56.89 | 5.83 |
| OEA_RS01705 | OEA_RS01705 | superoxide dismutase family protein | 20.48 | 4.36 |
| OEA_RS09190 | OEA_RS09190 | tyrosinase family protein | 16.51 | 4.05 |
| OEA_RS26625 | bofA | pro-sigmaK processing inhibitor BofA | 9.42 | 3.24 |
| OEA_RS09110 | OEA_RS09110 | threonine synthase | 4.07 | 2.02 |
| OEA_RS06915 | OEA_RS06915 | YjcZ family sporulation protein | 993.44 | 9.96 |
| OEA_RS05110 | OEA_RS05110 | YlmC/YmxH family sporulation protein | 47.39 | 5.57 |
| OEA_RS05490 | fliE | flagellar hook-basal body complex protein FliE | 33.58 | 5.07 |
| OEA_RS05105 | sigG | RNA polymerase sporulation sigma factor SigG | 175.95 | 7.46 |
| OEA_RS12285 | OEA_RS12285 | cold-shock protein | 19.75 | 4.30 |
| OEA_RS09965 | OEA_RS09965 | GvpL/GvpF family gas vesicle protein | 10.25 | 3.36 |
| OEA_RS21545 | OEA_RS21545 | ATPase | 5.35 | 2.42 |
| OEA_RS04590 | OEA_RS04590 | spore maturation protein | 5.44 | 2.44 |
| OEA_RS09940 | OEA_RS09940 | gas vesicle protein | 4.08 | 2.03 |
| OEA_RS16940 | OEA_RS16940 | glucose-6-phosphate dehydrogenase | 4.24 | 2.08 |
| OEA_RS05485 | flgC | flagellar basal body rod protein FlgC | 61.88 | 5.95 |
| OEA_RS02895 | OEA_RS02895 | aliphatic sulfonate ABC transporter substrate-binding protein | 8.61 | 3.11 |
| OEA_RS12005 | OEA_RS12005 | sugar ABC transporter permease | 38.42 | 5.26 |
| OEA_RS13475 | OEA_RS13475 | Hsp20/alpha crystallin family protein | 8.43 | 3.08 |
| OEA_RS15060 | OEA_RS15060 | YjcZ family sporulation protein | 203.55 | 7.67 |
| OEA_RS02080 | OEA_RS02080 | cytochrome d ubiquinol oxidase subunit II | 5.24 | 2.39 |
| OEA_RS21640 | pgsC | poly-gamma-glutamate biosynthesis protein PgsC | 37.62 | 5.23 |
| OEA_RS04515 | OEA_RS04515 | stage V sporulation protein AB | 79.32 | 6.31 |
| OEA_RS05510 | fliI | flagellar protein export ATPase FliI | 6.59 | 2.72 |
| OEA_RS04120 | OEA_RS04120 | stage III sporulation protein SpoAB | 9.08 | 3.18 |
| OEA_RS08490 | OEA_RS08490 | small acid-soluble spore protein H | 22.22 | 4.47 |
| OEA_RS19540 | OEA_RS19540 | sugar ABC transporter permease | 8.46 | 3.08 |
| OEA_RS07635 | OEA_RS07635 | YjcZ family sporulation protein | 796.38 | 9.64 |
| OEA_RS02900 | OEA_RS02900 | ABC transporter permease | 15.75 | 3.98 |
| OEA_RS05555 | fliY | flagellar motor switch phosphatase FliY | 5.32 | 2.41 |
| OEA_RS11830 | OEA_RS11830 | ABC transporter permease | 4.14 | 2.05 |
| OEA_RS04570 | OEA_RS04570 | YjcZ family sporulation protein | 151.70 | 7.25 |
| OEA_RS06230 | OEA_RS06230 | CoA transferase subunit A | 48.03 | 5.59 |
| OEA_RS15225 | OEA_RS15225 | alpha/beta-type small acid-soluble spore protein | 11.76 | 3.56 |
| OEA_RS26660 | OEA_RS26660 | cysteine hydrolase | 4.27 | 2.09 |
| OEA_RS00535 | OEA_RS00535 | TetR/AcrR family transcriptional regulator | 7.61 | 2.93 |
| OEA_RS22025 | rbsC | ribose ABC transporter permease | 4.36 | 2.12 |
| OEA_RS06940 | OEA_RS06940 | amino acid permease | 8.54 | 3.09 |
| OEA_RS20360 | OEA_RS20360 | spore germination protein | 16.97 | 4.08 |
| OEA_RS02905 | ssuE | NADPH-dependent FMN reductase | 17.84 | 4.16 |
| OEA_RS07305 | OEA_RS07305 | YjcZ family sporulation protein | 383.06 | 8.58 |
| OEA_RS33590 | OEA_RS33590 | YjcZ family sporulation protein | 61.82 | 5.95 |
| OEA_RS06920 | OEA_RS06920 | YjcZ family sporulation protein | 8.18 | 3.03 |
| OEA_RS05515 | fliJ | flagellar biosynthesis chaperone FliJ | 5.81 | 2.54 |
| OEA_RS05505 | fliH | flagellar assembly protein FliH | 9.07 | 3.18 |
| OEA_RS01810 | OEA_RS01810 | hypothetical protein | 5.53 | 2.47 |
| OEA_RS18300 | OEA_RS18300 | cupredoxin domain-containing protein | 6.79 | 2.76 |
| OEA_RS20005 | OEA_RS20005 | YjcZ family sporulation protein | 93.85 | 6.55 |
| OEA_RS09970 | OEA_RS09970 | gas vesicle protein GvpR | 17.37 | 4.12 |
| OEA_RS18500 | OEA_RS18500 | spore germination protein | 5.87 | 2.55 |
| OEA_RS07640 | OEA_RS07640 | YjcZ family sporulation protein | 168.65 | 7.40 |
| OEA_RS09975 | gvpA | gas vesicle structural protein GvpA | 12.96 | 3.70 |
| OEA_RS15525 | OEA_RS15525 | endospore germination permease | 6.31 | 2.66 |
| OEA_RS04530 | spoVAE | stage V sporulation protein AE | 11.95 | 3.58 |
| OEA_RS09960 | OEA_RS09960 | gas vesicle protein GvpG | 14.43 | 3.85 |
| OEA_RS09945 | OEA_RS09945 | gas vesicle protein K | 4.29 | 2.10 |
| OEA_RS24285 | OEA_RS24285 | YjcZ family sporulation protein | 8.69 | 3.12 |
| OEA_RS05300 | OEA_RS05300 | stage V sporulation protein SpoVM | 9.63 | 3.27 |
| OEA_RS23790 | OEA_RS23790 | YjcZ family sporulation protein | 58.23 | 5.86 |
| OEA_RS04130 | spoIIIAD | stage III sporulation protein AD | 9.71 | 3.28 |
| OEA_RS04125 | spoIIIAC | stage III sporulation protein AC | 6.85 | 2.78 |
| OEA_RS05530 | flgD | flagellar hook assembly protein FlgD | 4.44 | 2.15 |
| OEA_RS04450 | mciZ | Z-ring formation inhibitor MciZ | 5.78 | 2.53 |

**Supplementary Table 6** Significantly down-regulated differential genes in CK vs NCTa comparison group

| Gene_id | Gene name | Gene description | FC | Log2FC |
| --- | --- | --- | --- | --- |
| OEA_RS19565 | OEA_RS19565 | response regulator transcription factor | 0.10 | -3.31 |
| OEA_RS24895 | msrA | peptide-methionine (S)-S-oxide reductase MsrA | 0.23 | -2.10 |
| OEA_RS19570 | OEA_RS19570 | sensor histidine kinase | 0.07 | -3.89 |
| OEA_RS12775 | OEA_RS12775 | TerD family protein | 0.08 | -3.66 |
| OEA_RS12770 | OEA_RS12770 | TerD family protein | 0.08 | -3.60 |
| OEA_RS12765 | OEA_RS12765 | TerD family protein | 0.08 | -3.70 |
| OEA_RS25805 | OEA_RS25805 | anti-sigma factor | 0.25 | -2.01 |
| OEA_RS17880 | OEA_RS17880 | HAMP domain-containing histidine kinase | 0.05 | -4.31 |
| OEA_RS20415 | OEA_RS20415 | aspartyl-phosphate phosphatase Spo0E family protein | 0.20 | -2.33 |
| OEA_RS21235 | OEA_RS21235 | efflux RND transporter permease subunit | 0.09 | -3.56 |
| OEA_RS20615 | OEA_RS20615 | MerR family transcriptional regulator | 0.20 | -2.31 |
| OEA_RS16460 | OEA_RS16460 | response regulator transcription factor | 0.04 | -4.68 |
| OEA_RS16455 | OEA_RS16455 | HAMP domain-containing histidine kinase | 0.05 | -4.38 |
| OEA_RS13745 | OEA_RS13745 | GNAT family N-acetyltransferase | 0.13 | -2.90 |
| OEA_RS01335 | OEA_RS01335 | HAMP domain-containing histidine kinase | 0.04 | -4.63 |
| OEA_RS01330 | OEA_RS01330 | response regulator transcription factor | 0.02 | -5.43 |
| OEA_RS20880 | OEA_RS20880 | VOC family protein | 0.10 | -3.32 |
| OEA_RS06300 | OEA_RS06300 | serine/threonine protein kinase | 0.24 | -2.05 |
| OEA_RS04695 | ypdA | YpdA family putative bacillithiol disulfide reductase | 0.24 | -2.05 |
| OEA_RS17885 | OEA_RS17885 | response regulator transcription factor | 0.02 | -5.54 |
| OEA_RS12230 | OEA_RS12230 | alkylhydroperoxidase | 0.06 | -3.99 |
| OEA_RS19490 | OEA_RS19490 | GNAT family N-acetyltransferase | 0.23 | -2.10 |
| OEA_RS15305 | OEA_RS15305 | GNAT family N-acetyltransferase | 0.19 | -2.42 |
| OEA_RS20635 | OEA_RS20635 | response regulator transcription factor | 0.16 | -2.66 |
| OEA_RS06085 | OEA_RS06085 | PAS domain S-box protein | 0.24 | -2.08 |
| OEA_RS09230 | OEA_RS09230 | LemA family protein | 0.21 | -2.26 |
| OEA_RS17905 | OEA_RS17905 | response regulator transcription factor | 0.05 | -4.36 |
| OEA_RS16120 | OEA_RS16120 | HAMP domain-containing histidine kinase | 0.20 | -2.33 |
| OEA_RS07170 | OEA_RS07170 | MerR family transcriptional regulator | 0.24 | -2.06 |
| OEA_RS32715 | OEA_RS32715 | MerR family transcriptional regulator | 0.16 | -2.66 |
| OEA_RS21685 | OEA_RS21685 | diguanylate cyclase | 0.15 | -2.73 |
| OEA_RS18430 | OEA_RS18430 | MMPL family transporter | 0.24 | -2.06 |
| OEA_RS31110 | OEA_RS31110 | aspartyl-phosphate phosphatase Spo0E family protein | 0.16 | -2.68 |
| OEA_RS11375 | OEA_RS11375 | class F sortase | 0.24 | -2.04 |
| OEA_RS31105 | OEA_RS31105 | HTH domain-containing protein | 0.23 | -2.10 |
| OEA_RS16125 | OEA_RS16125 | response regulator transcription factor | 0.17 | -2.58 |
| OEA_RS18095 | OEA_RS18095 | MerR family transcriptional regulator | 0.02 | -5.45 |
| OEA_RS06975 | OEA_RS06975 | HTH-type transcriptional regulator Hpr | 0.25 | -1.98 |
| OEA_RS19185 | OEA_RS19185 | DsbA family oxidoreductase | 0.21 | -2.24 |
| OEA_RS13045 | OEA_RS13045 | GNAT family N-acetyltransferase | 0.16 | -2.69 |
| OEA_RS18190 | OEA_RS18190 | mechanosensitive ion channel family protein | 0.18 | -2.46 |
| OEA_RS09920 | OEA_RS09920 | YafY family transcriptional regulator | 0.15 | -2.72 |
| OEA_RS13415 | OEA_RS13415 | Asp23/Gls24 family envelope stress response protein | 0.21 | -2.23 |
| OEA_RS21805 | OEA_RS21805 | LysR family transcriptional regulator | 0.23 | -2.12 |
| OEA_RS25700 | OEA_RS25700 | response regulator transcription factor | 0.21 | -2.26 |
| OEA_RS21375 | OEA_RS21375 | EAL domain-containing protein | 0.19 | -2.43 |
| OEA_RS30200 | OEA_RS30200 | IS3 family transposase | 0.06 | -3.96 |
| OEA_RS06725 | OEA_RS06725 | GNAT family N-acetyltransferase | 0.22 | -2.18 |
| OEA_RS05960 | OEA_RS05960 | MerR family transcriptional regulator | 0.24 | -2.09 |
| OEA_RS13340 | OEA_RS13340 | GNAT family N-acetyltransferase | 0.18 | -2.44 |
| OEA_RS21675 | OEA_RS21675 | diguanylate cyclase | 0.18 | -2.50 |
| OEA_RS32485 | OEA_RS32485 | IS3 family transposase | 0.09 | -3.48 |
| OEA_RS18030 | OEA_RS18030 | tetracycline resistance MFS efflux pump | 0.24 | -2.06 |

**Supplementary Table 7** Significantly up-regulated differential genes in NCTa vsNCTb comparison group

| Gene_id | Gene name | Gene description | FC | Log2FC |
| --- | --- | --- | --- | --- |
| OEA_RS04150 | OEA_RS04150 | SpoIIIAH-like family protein | 10.03 | 3.33 |
| OEA_RS24005 | OEA_RS24005 | SpoVR family protein | 7.24 | 2.86 |
| OEA_RS02950 | OEA_RS02950 | sporulation protein | 7.67 | 2.94 |
| OEA_RS00420 | OEA_RS00420 | cell wall hydrolase | 6.25 | 2.64 |
| OEA_RS04145 | spoIIIAG | stage III sporulation protein AG | 11.00 | 3.46 |
| OEA_RS04140 | spoIIIAF | stage III sporulation protein AF | 13.27 | 3.73 |
| OEA_RS04115 | spoIIIAA | stage III sporulation protein AA | 13.63 | 3.77 |
| OEA_RS04075 | OEA_RS04075 | patatin-like phospholipase family protein | 7.77 | 2.96 |
| OEA_RS09900 | OEA_RS09900 | hypothetical protein | 12.30 | 3.62 |
| OEA_RS23520 | OEA_RS23520 | M48 family metallopeptidase | 8.77 | 3.13 |
| OEA_RS05940 | spoVK | stage V sporulation protein K | 7.88 | 2.98 |
| OEA_RS25890 | pdaB | polysaccharide deacetylase family sporulation protein PdaB | 7.16 | 2.84 |
| OEA_RS02700 | OEA_RS02700 | sporulation protein | 13.83 | 3.79 |
| OEA_RS16020 | OEA_RS16020 | superoxide dismutase family protein | 12.86 | 3.68 |
| OEA_RS31445 | OEA_RS31445 | alpha/beta-type small acid-soluble spore protein | 11.20 | 3.49 |
| OEA_RS06760 | OEA_RS06760 | two-component sensor histidine kinase | 5.82 | 2.54 |
| OEA_RS18910 | OEA_RS18910 | general stress protein | 5.96 | 2.57 |
| OEA_RS08555 | OEA_RS08555 | C40 family peptidase | 8.21 | 3.04 |
| OEA_RS19635 | OEA_RS19635 | cold-shock protein | 7.74 | 2.95 |
| OEA_RS01575 | yutH | spore coat protein YutH | 4.87 | 2.28 |
| OEA_RS05050 | OEA_RS05050 | stage V sporulation protein D | 6.23 | 2.64 |
| OEA_RS00730 | OEA_RS00730 | M23 family metallopeptidase | 8.16 | 3.03 |
| OEA_RS17460 | OEA_RS17460 | cell wall hydrolase | 15.49 | 3.95 |
| OEA_RS03075 | spoVID | stage VI sporulation protein D | 7.34 | 2.88 |
| OEA_RS09895 | OEA_RS09895 | spore coat protein CotJB | 9.58 | 3.26 |
| OEA_RS05880 | spoVS | stage V sporulation protein SpoVS | 6.53 | 2.71 |
| OEA_RS31165 | OEA_RS31165 | cell wall hydrolase | 7.91 | 2.98 |
| OEA_RS32820 | OEA_RS32820 | Ger(x)C family spore germination protein | 8.04 | 3.01 |
| OEA_RS21155 | OEA_RS21155 | flagellin | 12.22 | 3.61 |
| OEA_RS04120 | OEA_RS04120 | stage III sporulation protein SpoAB | 10.68 | 3.42 |
| OEA_RS03230 | safA | SafA/ExsA family spore coat assembly protein | 6.20 | 2.63 |
| OEA_RS10125 | OEA_RS10125 | manganese catalase family protein | 5.44 | 2.44 |
| OEA_RS18325 | OEA_RS18325 | cold-shock protein | 4.23 | 2.08 |
| OEA_RS21160 | OEA_RS21160 | flagellin | 16.06 | 4.01 |
| OEA_RS32815 | OEA_RS32815 | spore germination protein | 7.21 | 2.85 |
| OEA_RS15810 | OEA_RS15810 | squalene--hopene cyclase | 5.93 | 2.57 |
| OEA_RS06955 | OEA_RS06955 | GerAB/ArcD/ProY family transporter | 5.70 | 2.51 |
| OEA_RS04780 | spoIVA | stage IV sporulation protein A | 6.16 | 2.62 |
| OEA_RS03295 | spoVB | stage V sporulation protein B | 7.44 | 2.90 |
| OEA_RS18005 | OEA_RS18005 | spore germination protein | 6.98 | 2.80 |
| OEA_RS13385 | OEA_RS13385 | multidrug efflux MFS transporter | 4.63 | 2.21 |
| OEA_RS09890 | OEA_RS09890 | spore coat associated protein CotJA | 10.53 | 3.40 |
| OEA_RS11310 | OEA_RS11310 | Hsp20/alpha crystallin family protein | 5.20 | 2.38 |
| OEA_RS02570 | OEA_RS02570 | sporulation protein | 7.48 | 2.90 |
| OEA_RS26625 | bofA | pro-sigmaK processing inhibitor BofA | 7.95 | 2.99 |
| OEA_RS05070 | spoVE | stage V sporulation protein E | 5.81 | 2.54 |
| OEA_RS05105 | sigG | RNA polymerase sporulation sigma factor SigG | 4.60 | 2.20 |
| OEA_RS24460 | yhbH | sporulation protein YhbH | 4.15 | 2.05 |
| OEA_RS31120 | OEA_RS31120 | chromosome partitioning protein ParA | 20.58 | 4.36 |
| OEA_RS31145 | OEA_RS31145 | SOS response-associated peptidase | 7.32 | 2.87 |
| OEA_RS02495 | ytfJ | sporulation protein YtfJ | 5.23 | 2.39 |
| OEA_RS04135 | spoIIIAE | stage III sporulation protein AE | 17.09 | 4.10 |
| OEA_RS03155 | OEA_RS03155 | M23 family metallopeptidase | 6.00 | 2.59 |
| OEA_RS12285 | OEA_RS12285 | cold-shock protein | 7.23 | 2.85 |
| OEA_RS03160 | OEA_RS03160 | stage IV sporulation protein FB | 6.63 | 2.73 |
| OEA_RS06790 | OEA_RS06790 | TetR/AcrR family transcriptional regulator | 11.82 | 3.56 |
| OEA_RS00200 | OEA_RS00200 | HAMP domain-containing histidine kinase | 9.87 | 3.30 |
| OEA_RS33360 | OEA_RS33360 | chromosome partitioning protein ParA | 6.42 | 2.68 |
| OEA_RS04960 | ylbJ | sporulation integral membrane protein YlbJ | 8.82 | 3.14 |
| OEA_RS04590 | OEA_RS04590 | spore maturation protein | 7.53 | 2.91 |
| OEA_RS09910 | OEA_RS09910 | TetR/AcrR family transcriptional regulator | 5.90 | 2.56 |
| OEA_RS26370 | yabP | sporulation protein YabP | 5.14 | 2.36 |
| OEA_RS04595 | OEA_RS04595 | spore maturation protein | 8.36 | 3.06 |
| OEA_RS00415 | gerQ | spore coat protein GerQ | 5.21 | 2.38 |
| OEA_RS18285 | OEA_RS18285 | H-type small acid-soluble spore protein | 4.52 | 2.17 |
| OEA_RS02610 | ytvI | sporulation integral membrane protein YtvI | 5.83 | 2.54 |
| OEA_RS20595 | OEA_RS20595 | Ger(x)C family spore germination protein | 7.38 | 2.88 |
| OEA_RS23680 | OEA_RS23680 | TetR/AcrR family transcriptional regulator | 5.23 | 2.39 |
| OEA_RS19280 | OEA_RS19280 | spore germination protein | 0.27 | -1.89 |
| OEA_RS09965 | OEA_RS09965 | GvpL/GvpF family gas vesicle protein | 5.23 | 2.39 |
| OEA_RS24800 | OEA_RS24800 | winged helix-turn-helix transcriptional regulator | 4.29 | 2.10 |
| OEA_RS15225 | OEA_RS15225 | alpha/beta-type small acid-soluble spore protein | 6.33 | 2.66 |
| OEA_RS22840 | OEA_RS22840 | TetR/AcrR family transcriptional regulator | 6.12 | 2.61 |
| OEA_RS16515 | motA | flagellar motor stator protein MotA | 6.92 | 2.79 |
| OEA_RS01310 | OEA_RS01310 | MarR family transcriptional regulator | 11.79 | 3.56 |
| OEA_RS18000 | OEA_RS18000 | Ger(x)C family spore germination protein | 4.22 | 2.08 |
| OEA_RS32825 | OEA_RS32825 | GerAB/ArcD/ProY family transporter | 4.90 | 2.29 |
| OEA_RS14670 | OEA_RS14670 | MarR family transcriptional regulator | 10.61 | 3.41 |
| OEA_RS33200 | OEA_RS33200 | chromosome partitioning protein ParA | 5.44 | 2.44 |
| OEA_RS04130 | spoIIIAD | stage III sporulation protein AD | 12.56 | 3.65 |
| OEA_RS10485 | OEA_RS10485 | endospore germination permease | 4.89 | 2.29 |
| OEA_RS06795 | OEA_RS06795 | DHA2 family efflux MFS transporter permease subunit | 4.46 | 2.16 |
| OEA_RS04460 | spoIIM | stage II sporulation protein M | 5.45 | 2.45 |
| OEA_RS04125 | spoIIIAC | stage III sporulation protein AC | 9.88 | 3.30 |
| OEA_RS12180 | OEA_RS12180 | Ger(x)C family spore germination protein | 4.76 | 2.25 |
| OEA_RS01810 | OEA_RS01810 | hypothetical protein | 4.06 | 2.02 |
| OEA_RS04450 | mciZ | Z-ring formation inhibitor MciZ | 11.63 | 3.54 |
| OEA_RS33380 | OEA_RS33380 | helix-turn-helix transcriptional regulator | 6.27 | 2.65 |
| OEA_RS06455 | OEA_RS06455 | GerAB/ArcD/ProY family transporter | 6.94 | 2.79 |
| OEA_RS09975 | gvpA | gas vesicle structural protein GvpA | 4.69 | 2.23 |
| OEA_RS21165 | OEA_RS21165 | flagellin B | 16.75 | 4.07 |
| OEA_RS09970 | OEA_RS09970 | gas vesicle protein GvpR | 4.42 | 2.14 |
| OEA_RS18880 | OEA_RS18880 | alpha/beta-type small acid-soluble spore protein | 2.08 | 1.06 |
| OEA_RS31360 | OEA_RS31360 | histidine phosphatase family protein | 7.11 | 2.83 |
| OEA_RS07265 | OEA_RS07265 | stage II sporulation protein P | 7.06 | 2.82 |
| OEA_RS17530 | OEA_RS17530 | GNAT family N-acetyltransferase | 4.06 | 2.02 |
| OEA_RS10520 | OEA_RS10520 | MarR family transcriptional regulator | 5.75 | 2.52 |
| OEA_RS17990 | OEA_RS17990 | GerAB/ArcD/ProY family transporter | 4.82 | 2.27 |
| OEA_RS05300 | OEA_RS05300 | stage V sporulation protein SpoVM | 4.73 | 2.24 |
| OEA_RS17985 | OEA_RS17985 | GerAB/ArcD/ProY family transporter | 5.09 | 2.35 |
| OEA_RS19160 | OEA_RS19160 | DHA2 family efflux MFS transporter permease subunit | 11.60 | 3.54 |
| OEA_RS02250 | OEA_RS02250 | sporulation protein Cse60 | 5.81 | 2.54 |
| OEA_RS15610 | OEA_RS15610 | GerAB/ArcD/ProY family transporter | 4.13 | 2.05 |
| OEA_RS19155 | OEA_RS19155 | MarR family transcriptional regulator | 5.66 | 2.50 |
| OEA_RS19150 | OEA_RS19150 | helix-turn-helix transcriptional regulator | 4.34 | 2.12 |
| OEA_RS17870 | OEA_RS17870 | sporulation histidine kinase inhibitor Sda | 5.06 | 2.34 |
| OEA_RS33370 | OEA_RS33370 | aspartyl-phosphate phosphatase Spo0E family protein | 4.57 | 2.19 |

**Supplementary Table 8** Significantly down-regulated differential genes in NCTa vsNCTb comparison group

| Gene_id | Gene name | Gene description | FC | Log2FC |
| --- | --- | --- | --- | --- |
| OEA_RS10380 | qoxB | cytochrome aa3 quinol oxidase subunit I | 0.14 | -2.82 |
| OEA_RS10375 | qoxA | cytochrome aa3 quinol oxidase subunit II | 0.12 | -3.10 |
| OEA_RS19055 | OEA_RS19055 | betaine/proline/choline family ABC transporter ATP-binding protein | 0.04 | -4.78 |
| OEA_RS19120 | OEA_RS19120 | ABC transporter ATP-binding protein | 0.07 | -3.82 |
| OEA_RS19125 | OEA_RS19125 | iron-hydroxamate ABC transporter substrate-binding protein | 0.08 | -3.58 |
| OEA_RS00295 | cydD | thiol reductant ABC exporter subunit CydD | 0.02 | -5.74 |
| OEA_RS00290 | cydB | cytochrome d ubiquinol oxidase subunit II | 0.02 | -5.82 |
| OEA_RS00285 | OEA_RS00285 | cytochrome ubiquinol oxidase subunit I | 0.02 | -5.99 |
| OEA_RS02140 | OEA_RS02140 | ABC transporter permease | 0.12 | -3.01 |
| OEA_RS19050 | OEA_RS19050 | proline/glycine betaine ABC transporter permease | 0.03 | -4.89 |
| OEA_RS02150 | OEA_RS02150 | ABC transporter substrate-binding protein | 0.13 | -2.95 |
| OEA_RS19385 | OEA_RS19385 | ABC transporter ATP-binding protein | 0.09 | -3.43 |
| OEA_RS02145 | OEA_RS02145 | ABC transporter ATP-binding protein | 0.12 | -3.03 |
| OEA_RS03050 | OEA_RS03050 | cytochrome c biogenesis protein CcsA | 0.10 | -3.31 |
| OEA_RS04610 | OEA_RS04610 | cytochrome c biogenesis protein | 0.21 | -2.24 |
| OEA_RS22295 | OEA_RS22295 | ABC transporter substrate-binding protein | 0.24 | -2.06 |
| OEA_RS10385 | qoxC | cytochrome aa3 quinol oxidase subunit III | 0.17 | -2.60 |
| OEA_RS23890 | ugpC | sn-glycerol-3-phosphate ABC transporter ATP-binding protein UgpC | 0.19 | -2.42 |
| OEA_RS19395 | OEA_RS19395 | ABC transporter permease | 0.22 | -2.17 |
| OEA_RS00300 | cydC | thiol reductant ABC exporter subunit CydC | 0.16 | -2.62 |
| OEA_RS19390 | OEA_RS19390 | ABC transporter permease | 0.14 | -2.86 |
| OEA_RS04615 | ccsB | c-type cytochrome biogenesis protein CcsB | 0.22 | -2.18 |
| OEA_RS11845 | OEA_RS11845 | ABC transporter ATP-binding protein | 0.23 | -2.14 |
| OEA_RS11825 | OEA_RS11825 | ABC transporter substrate-binding protein | 0.21 | -2.27 |
| OEA_RS19130 | OEA_RS19130 | iron ABC transporter permease | 0.16 | -2.66 |
| OEA_RS24140 | OEA_RS24140 | ABC transporter substrate-binding protein | 0.25 | -2.02 |
| OEA_RS17205 | OEA_RS17205 | cytochrome c oxidase subunit II | 0.19 | -2.37 |
| OEA_RS19530 | ugpC | sn-glycerol-3-phosphate ABC transporter ATP-binding protein UgpC | 0.18 | -2.45 |
| OEA_RS17200 | qoxB | cytochrome aa3 quinol oxidase subunit I | 0.24 | -2.07 |
| OEA_RS19535 | OEA_RS19535 | extracellular solute-binding protein | 0.20 | -2.34 |
| OEA_RS16105 | OEA_RS16105 | sulfate ABC transporter substrate-binding protein | 0.23 | -2.10 |
| OEA_RS02895 | OEA_RS02895 | aliphatic sulfonate ABC transporter substrate-binding protein | 0.18 | -2.48 |
| OEA_RS19540 | OEA_RS19540 | sugar ABC transporter permease | 0.15 | -2.77 |
| OEA_RS11830 | OEA_RS11830 | ABC transporter permease | 0.24 | -2.06 |
| OEA_RS10390 | qoxD | cytochrome aa3 quinol oxidase subunit IV | 0.16 | -2.63 |
| OEA_RS16395 | OEA_RS16395 | amino acid ABC transporter permease | 0.18 | -2.52 |
| OEA_RS11400 | OEA_RS11400 | branched-chain amino acid ABC transporter permease | 0.19 | -2.43 |

**Supplementary Table 9** Statistical table for evaluation of ribosomal RNA contamination rate

| Sample Name | rRNA Ratio(%) of Rfam |
| --- | --- |
| CK1 | 0.156 |
| CK2 | 0.156 |
| CK3 | 0.189 |
| NCTa1 | 0.354 |
| NCTa2 | 0.483 |
| NCTa3 | 0.31 |
| NCTb1 | 0.219 |
| NCTb2 | 0.34 |
| NCTb3 | 0.365 |
